# First stage in technological production of Stone Age animal teeth pendants: evidence from Zvejnieki (Latvia) and wider social implications

**DOI:** 10.1007/s12520-025-02260-0

**Published:** 2025-06-20

**Authors:** Aija Macāne, Kerkko Nordqvist, Kristiina Mannermaa, Andy Needham, Diederik Pomstra, Gabriel Cifuentes Alcobendas, Jānis Reblis, Ilga Zagorska, Aimée Little

**Affiliations:** 1 Department of Cultures, University of Helsinki, Helsinki, Finland; 2 Institute of Latvian History, University of Latvia, Riga, Latvia; 3 Helsinki Collegium for Advanced Studies, University of Helsinki, Helsinki, Finland; 4 Centre for Artefacts and Materials Analysis, Department of Archaeology, University of York, York, UK; 5 Faculty of Archaeology, Leiden University, Leiden, the Netherlands; 6 Department of History and Philosophy, University of Alcala, Alcalá de Henares, Spain; 7 Independent Participant, Īdeņa, Latvia

**Keywords:** Stone Age, Hunter-gatherers, Animal teeth, Personal ornaments, *Chaîne opératoire*, Experimental archaeology

## Abstract

Animal teeth were amongst the most common materials utilised for personal ornament production during the Stone Age, especially in the Northern Hemisphere. The hunter-gatherer cemetery site of Zvejnieki (Latvia) (7500–2500 cal. BC) is a clear example of this, with more than 2000 animal teeth excavated from grave contexts. Animal tooth pendants from the site have received significant scholarly attention, largely focusing on their faunal identification, type of use, placement within graves, as well as aspects of their production. Considerably less attention, however, has been given to the process of extracting teeth and the corresponding physical traces this might leave behind. This is true for Zvejnieki, but also for teeth extraction for the purposes of personal ornamentation across early prehistory more generally. To address this gap, we have employed experimental archaeological methods to critically evaluate possible Stone Age techniques of tooth extraction from key ungulate species, assessing the diagnostic traces created on the tooth itself and on the skull or mandible. The results suggest that several different methods of tooth extraction are viable, but cooking animals using boiling or a pit method is highly efficient. These methods lead to high extraction rates with no tooth damage, while also rendering the meat from the skull edible and the bones usable for other applications, such as tool production. Our research provides insights into the relationships between different spheres of hunter-gatherer life and death at Zvejnieki, specifically the acquisition of game animals, their treatment, and how this interacts with the extraction and processing of materials for craftwork and food preparation.

**Supplementary Information:**

The online version contains supplementary material available at 10.1007/s12520-025-02260-0.

## Introduction

Animal remains, particularly teeth, were one of the most common materials used for making personal ornaments during the Stone Age (from the Palaeolithic to the Neolithic), especially in northern Europe (e.g. Albrehtsen and Brinch Petersen 1977; Grünberg 2000, 2013; Gurina 1956; Janzon 1974; Larsson 1988; Macāne 2022; Mannermaa et al. 2021; Zagorskis 1987). Teeth utilised as a material for the crafting of pendants during the Holocene in hunter-gatherer contexts stem from a diverse array of species. Depending on the environmental setting, different mammals, including humans, ungulates (aurochs, bison, elk, red deer, reindeer, wild boar, roe deer, fallow deer, wild horse), carnivores (bear, dog, wolf, fox, badger, pine marten, otter, wild cat, seal) and rodents (beaver, marmot) have been used in northern Europe and beyond. Animal tooth pendants have attracted a range of theoretical and methodological studies (e.g., Bar-Yosef Mayer and Bosch 2019; Bar-Yosef Mayer et al. 2017; Borić and Cristiani 2019; Laporte and Dupont 2019; Macāne et al. 2019; Osipowicz et al. 2020, 2023; Rigaud 2011, 2013; Rigaud et al. 2013, 2019; Rainio and Mannermaa 2014; Rainio et al. 2021 and see below).

**Fig. 1:**
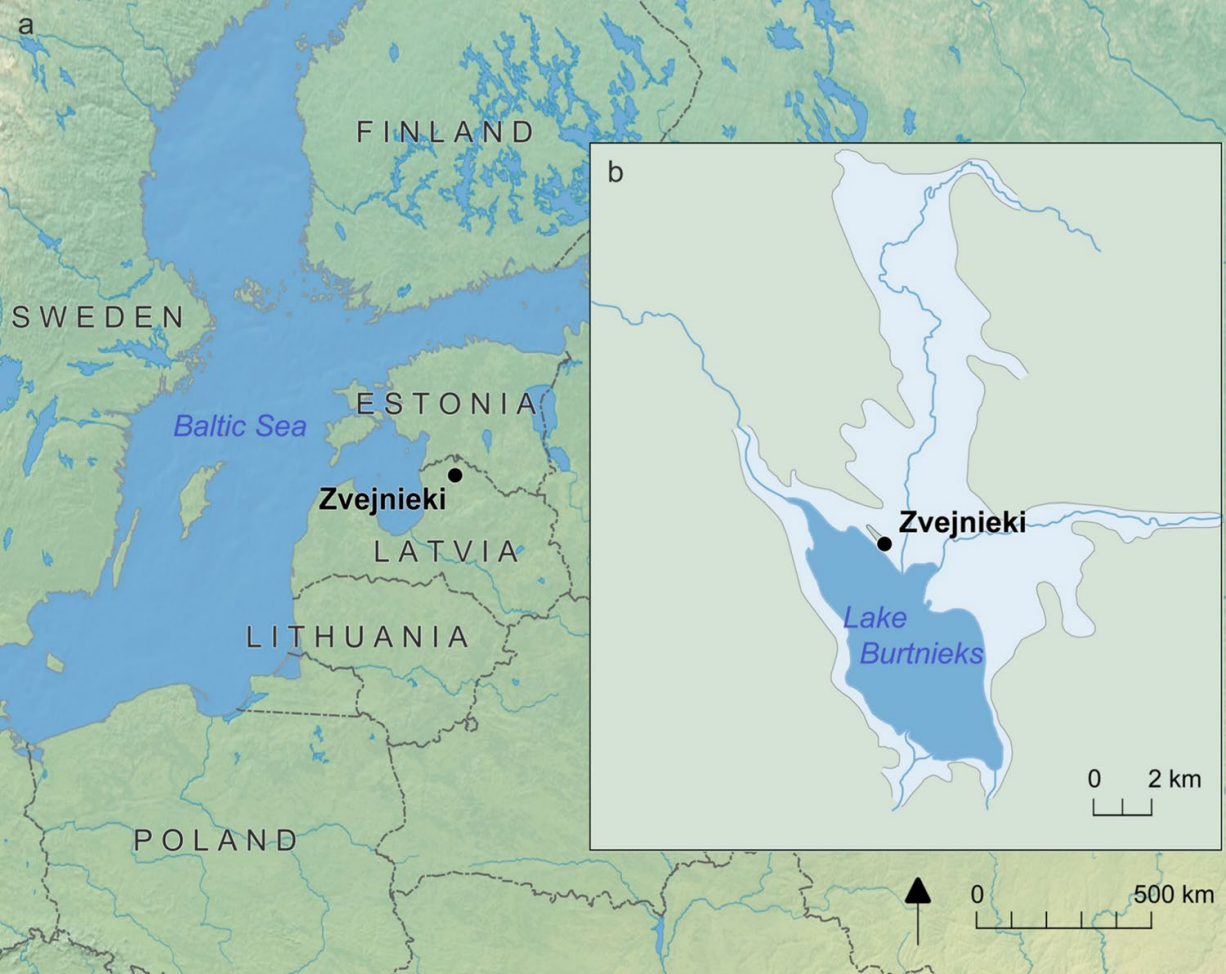
Map showing the location of Zvejnieki in northern Latvia, on the northern shore of Lake Burtnieks. The extent of the current lake is dark blue; Burtnieks palaeolake is light blue. Map by K. Nordqvist (after Eberhards 2006)

The well-known hunter-gatherer cemetery at Zvejnieki (Fig. 1) in northern Latvia provides one of the largest collections of animal teeth found in burials in north-eastern Europe, comprising more than two thousand examples including both modified and unmodified teeth. Of the 330 burials excavated at Zvejnieki, 115 contain grave goods, representing around one third of the total. Animal remains (mainly teeth) are present in more than 80% of these burials (Macāne 2022). The animal tooth pendants from the Zvejnieki burials have been discussed in several publications, primarily concerning their production, use and deposition, as well as taxonomic composition of the animal species, alongside their aesthetics and symbolism (David 2006; Larsson 2006, 2012, 2020; Lõugas 2006; Macāne 2022; Osipowicz et al. 2023; Zagorska and Lõugas 2000; Zagorskis 1987). Yet, the methods for extracting teeth from mandibles—a key stage in the production of ornaments—have rarely been considered: neither at Zvejnieki and other large hunter-gatherer cemeteries where similar pendants have been found (e.g., Albrehtsen and Brinch Petersen 1977; Butrimas 2012; Guminski 2014; Gurina 1956; Larsson 1988, 2016), nor in settlement contexts. This key gap in knowledge is curious considering a *chaîne opératoire* methodology has been routinely used to discuss the production of other types of artefacts from this site, including lithic and osseous tools (Berg-Hansen et al. 2019; Damlien et al. 2018; David 2003, 2006; Petrović et al. 2024). Lack of attention to this important stage of teeth pendant *chaîne opératoire* has arguably created a perception that teeth come ready for modification as pre-forms, disconnected from their animal source. As such, we are missing a vital part of the life history of these artefacts and how their manufacture was bound up with complex human-animal interactions and negotiations.

To fill this gap, this paper presents the results of a series of experiments designed to test different methods of tooth extraction from three common animal species found at Zvejnieki: Eurasian elk (*Alces alces*)*,* wild boar (*Sus scrofa*) and red deer (*Cervus elaphus*). We included roe deer (*Capreolus capreolus*) as a proxy for red deer, because red deer were not locally available at the time the experiments were undertaken. Drawing on observations from the analysis of archaeological teeth pendants from the Zvejnieki cemetery and experimental archaeology results, we present a range of possible methods of teeth extraction, thus providing new insights into this understudied stage in teeth pendant manufacture. We suggest that animal teeth extraction may, in some instances, have been linked to cooking and consumption practices. These results open up new lines of inquiry on the economic and social organisation of teeth pendant production and at the same time enable a deeper understanding of the various human–human and human–non-human interactions experienced during this key technological phase.

## Zvejnieki cemetery site context and animal teeth inventory

Zvejnieki is located on a former island in Lake Burtnieks, northern Latvia (Fig. 1). The majority of burials are dated between 7500 and 2500 cal. BC, with a small number of more recent burials (Meadows et al. 2018; Zagorska 2006; Zagorska et al. 2018). The cemetery is surrounded by settlement areas belonging to both the Mesolithic and Neolithic periods according to the Stone Age periodisation in north-eastern Europe. In the eastern Baltic region, pottery introduction marks the shift between the Mesolithic and the Neolithic, however, hunting, fishing, fowling and gathering formed a subsistence base throughout the Stone Age. Hence, *Stone Age* rather than Mesolithic/Neolithic, is the term used here. Zvejnieki was intensively excavated during the 1960 s and 1970 s (Zagorskis 1987; Zagorska 2016, 2019), with new excavations taking place in the early 2000 s (Larsson et al. 2017).

**Fig. 2:**
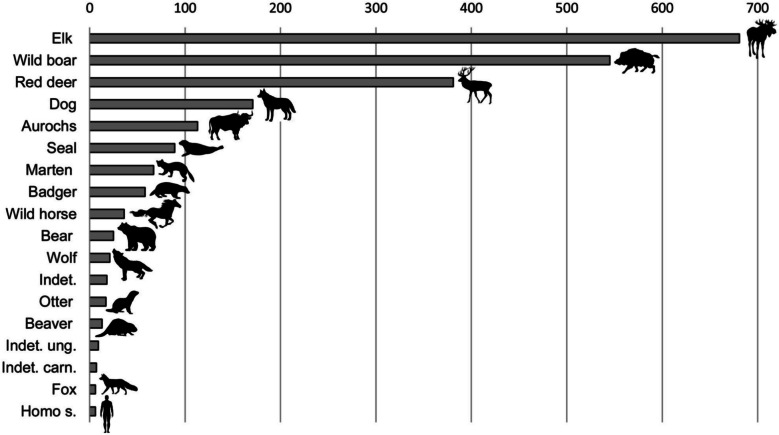
Number of animal teeth present in hunter-gatherer burials with secure contexts at the Zvejnieki cemetery. Illustration by K. Nordqvist based on data from Macāne 2022

**Fig. 3:**
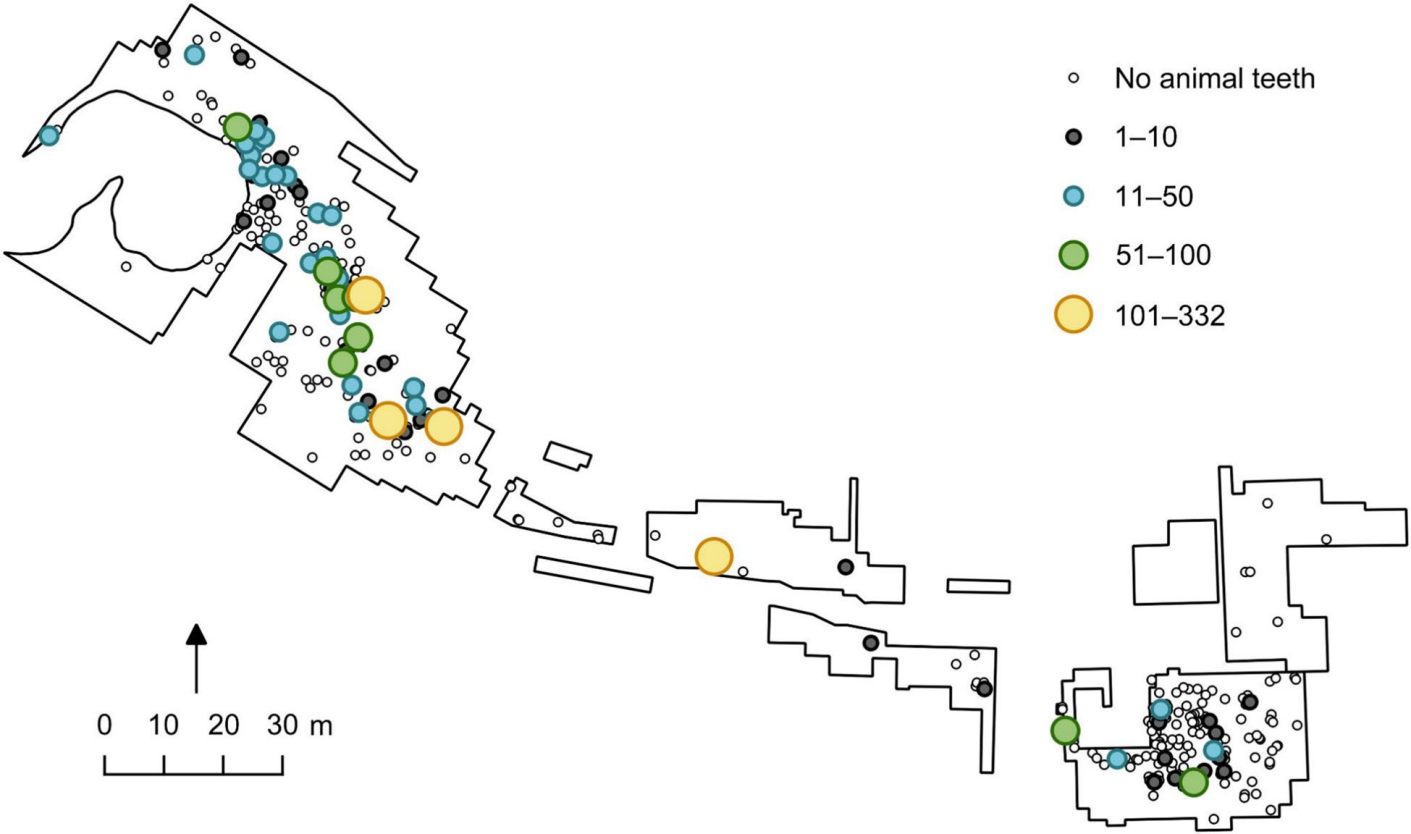
Distribution of burials with animal teeth at Zvejnieki, showing the number of animal teeth present in secure hunter-gatherer burial contexts. Illustration by K. Nordqvist (after Macāne 2022)

Within the cemetery, among the personal ornaments made from animal teeth, Eurasian elk teeth comprise the largest number (n. 681), followed by wild boar (n. 545). Teeth from ungulate species such as red deer and aurochs (*Bos primigenius*) have also been used for making pendants, but in fewer numbers, n. 381 and n. 113 respectively (Fig. 2). All these species are most common during the early stages of the use of the cemetery, while in the later phases, pendants are made mostly from carnivore species, such as dog (*Canis familiaris*), seals (Phocidae) and pine marten (*Martes martes*) (Macāne 2022). In most burials, the number of animal teeth varies from less than 10 to several dozen, but usually does not exceed 50. Seven burials have more than 50 teeth; in only four burials the number exceeds 100 (Fig. 3).

**Fig. 4:**
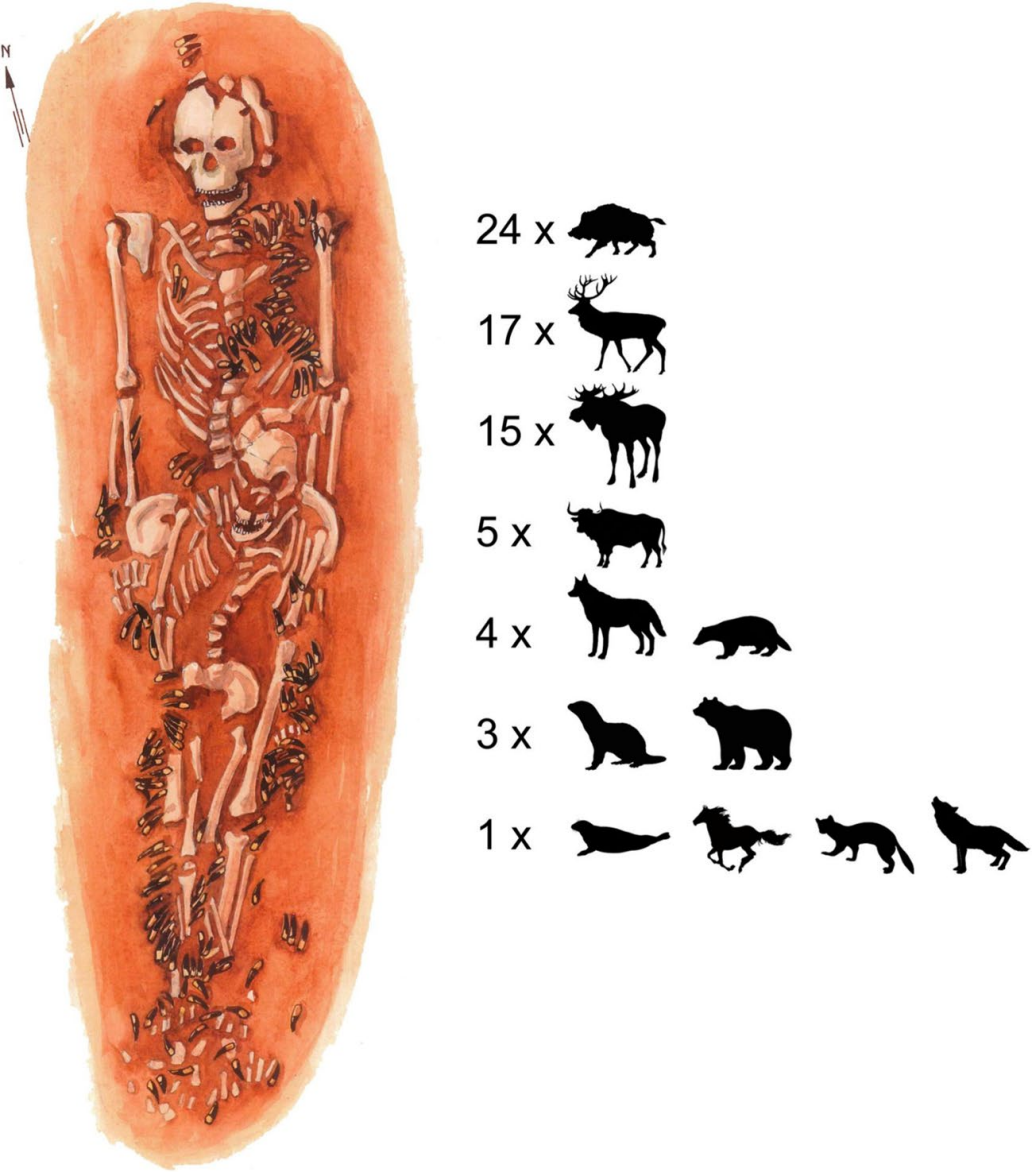
Minimum number of individuals of different species required to produce the animal tooth assemblage discovered in the double burial 122–123 at Zvejnieki. Illustration by K. Nordqvist (after Macāne 2022 and the grave drawing by B. Vaska, Institute of Latvian History, University of Latvia)

The largest quantity of teeth (n. 332) is found in a double burial 122–123. Teeth come from at least 24 wild boars, 17 red deer, 15 elks, 5 aurochs, 4 dogs, 4 badgers, 3 bears, 3 otters, as well as a pine marten, a seal, a wild horse (*Equus* sp.) and a wolf (*Canis lupus*) (Macāne 2022) (Fig. 4). The variety of animal species and their number suggest that significant time and effort was involved in their acquisition and processing. Large quantities of animal teeth pendants in some of the burials further suggest that the teeth were either acquired over a relatively long period of time, curated as raw material, and/or obtained via exchange networks (Brinch Petersen 2015; Cristiani and Borić 2012; Larsson 2006; Macāne et. al. 2023a). The presence of non-local artefacts such as seal teeth pendants perhaps demonstrates this point most clearly, indicating the movement of people and/or raw materials between the seacoast and Zvejnieki (Eriksson 2006).

Despite abundant studies on the animal teeth pendants from the Zvejnieki cemetery (see references above), the pendant production has never been approached from the point at which teeth were acquired and extracted from mandibles. Previous studies have shown that most of the teeth at Zvejnieki display anthropogenic traces (i.e., perforations, suspension grooves, fine grinding striations), but these relate to manufacture or wear, not extraction.

Little analytical work has been carried out on the faunal remains from the Zvejnieki settlement sites adjacent to the cemetery (Lõugas 2006; Sloka 1985; Zagorska 1992). More problematically, the faunal assemblage from the settlement contexts is incomplete due to the recovery-bias caused by selective recovery and excavation methods involving no sieving. Consequently, much of the small, unworked, fragmented faunal assemblage was never collected. Moreover, many of the bones were reburied directly after preliminary zooarchaeological identification, while the remaining collection has suffered from poor storage conditions, often resulting in the loss of original contextual information. As an exception, osseous tools and the related debitage were recovered more accurately and therefore understandably attracted more attention (David 2006; Macāne et al. 2023b).

Despite these preservation issues, it is clear that differences exist between the settlement and the cemetery in terms of the number, animal species, and technological choices surrounding the teeth pendant production. The settlement assemblage contains far fewer animal teeth pendants—approximately 130—compared to the over 2200 animal teeth recovered from burial contexts (nearly 2000 of them pendants). This supports an argument that teeth pendants may have been primarily intended for burials. Here, however, it is important to remember that depositions of personal ornaments at settlement sites are likely to be palimpsests of decorative items that served a variety of functions; some may simply have been lost or put aside for repair, etc. (Rigaud and Little 2025). To make a stronger case for the exclusive making of teeth pendants for funeral purposes would require use-wear analysis using high power microscopy to demonstrate that pendants displayed no traces of prior use. As these objects underwent conservation treatment several decades ago, a coating of preservation materials prevented such analysis. Furthermore, to date, there is no information on whether the mandibles from ungulates or other animals found during the excavation of the settlement display damage which can be linked to tooth extraction.

**Fig. 5:**
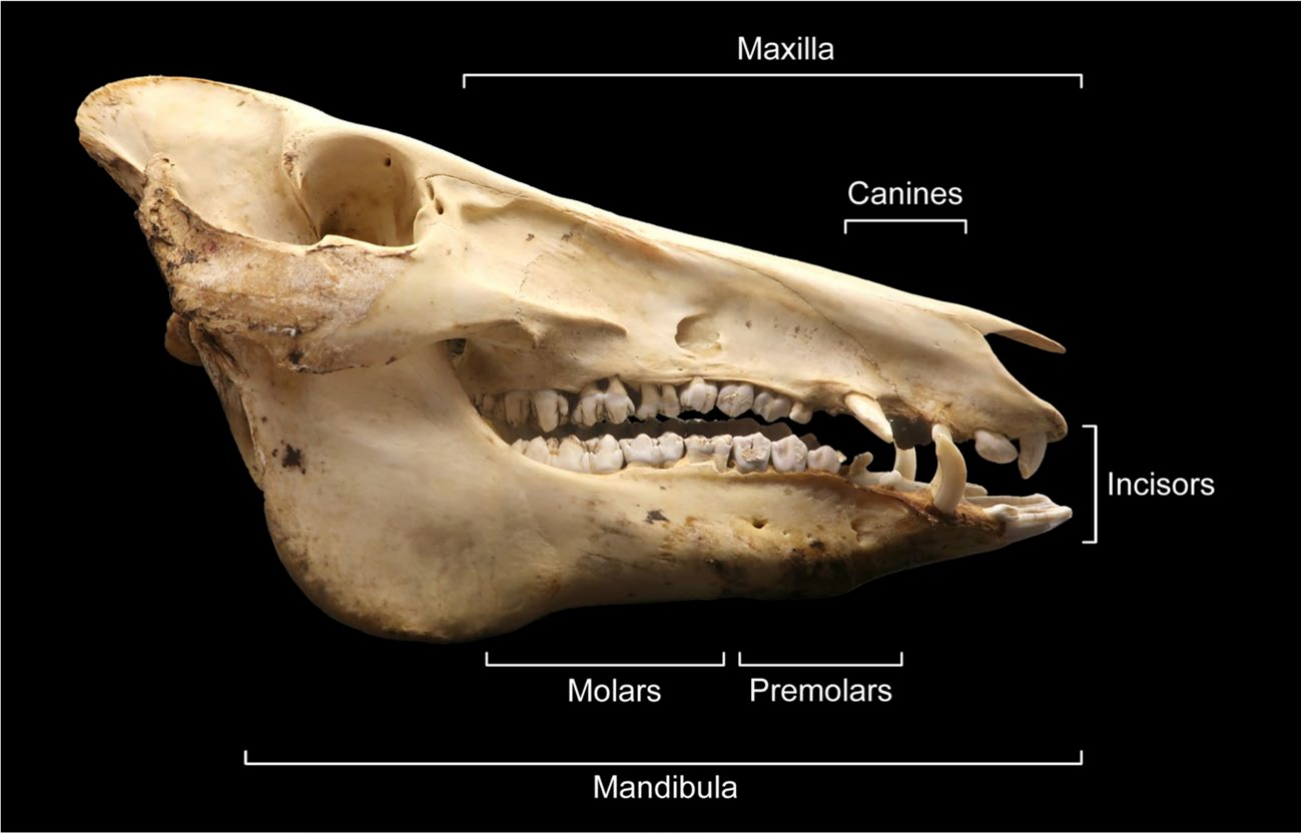
Lateral view of a wild boar skull, indicating the terminology for animal skull and teeth used in this article. Illustration by K. Nordqvist (terminology after Hillson 2005)

**Fig. 6:**
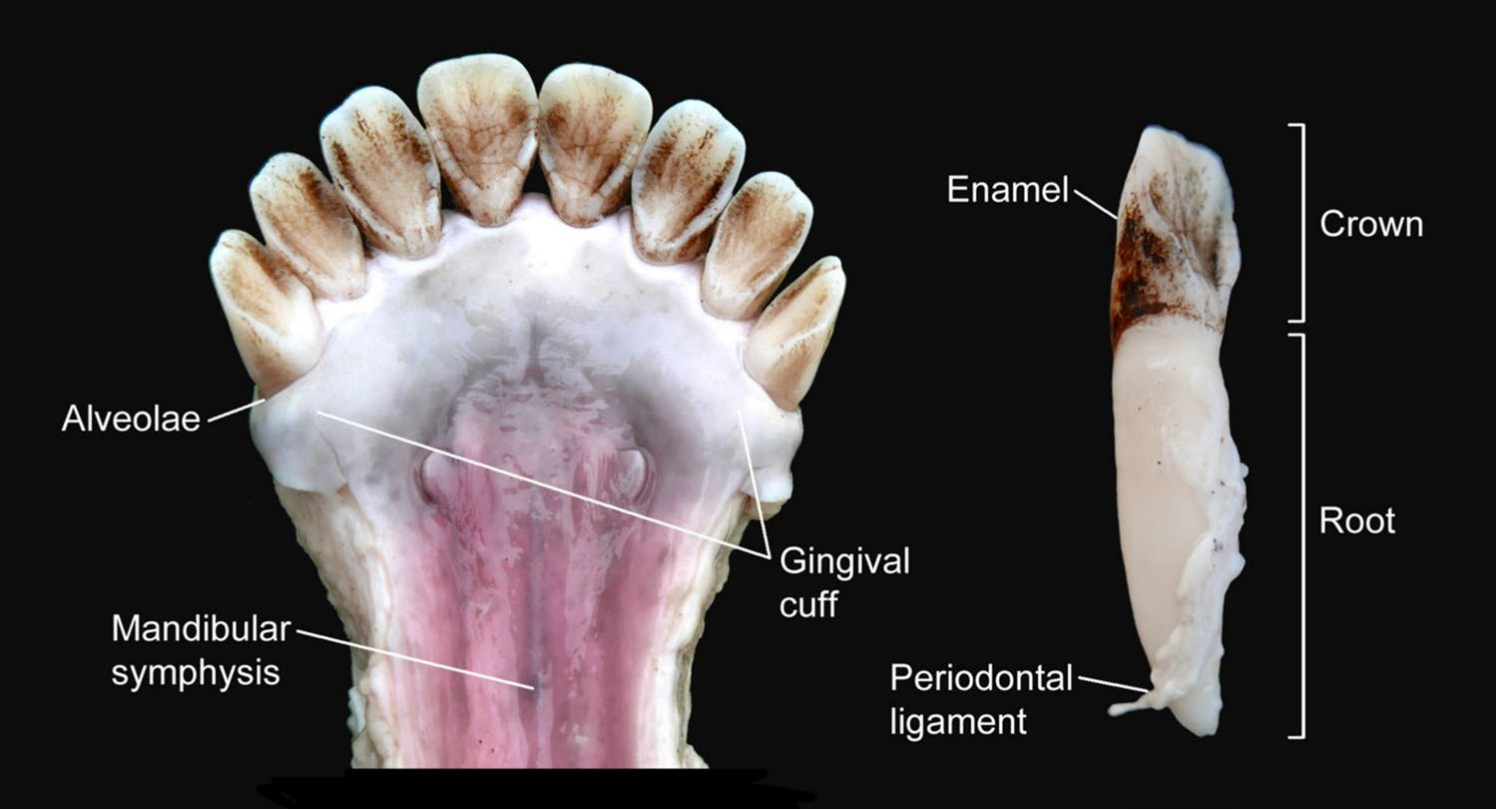
Eurasian elk mandibula and first right incisor with periodontal ligament still attached to the root. The photo taken during the soaking experiment (E4) illustrates key terminology related to teeth and the interior of mouth. Illustration by K. Nordqvist (terminology after Hillson 2005)

To gain a fuller understanding of *all* the technological stages of teeth pendant production at Zvejnieki, we carried out a series of actualistic (or as close to actualistic as possible) experiments, which are reported here. The primary aim was to test the viability of different extraction methods. Viability in this context is defined by the ability to remove teeth from the mandible without damage or extraction traces visible to the naked eye, thus replicating the absence of extraction-related traces on the archaeological specimens. The experiments targeted the main elements and species encountered at Zvejnieki: elk incisors, roe deer incisors (as a proxy for red deer), and wild boar incisors and canines (see Figs. 5 and 6 for an explanation of the skull and teeth terminology used). As these species are commonly found at other Stone Age burial sites in north-eastern Europe, our research has wider implications for understanding the manufacturing processes of animal teeth pendants beyond Zvejnieki.

## Materials and methods

Seven experimental methods (E1–7) were tested as a means of understanding the extraction phase of teeth pendant production at Zvejnieki. Below we provide a hypothesis and rationale for the choice of each experimental method, an overview of the methods/materials used, and a short discussion of the results. The discussion that follows considers the broader implications of the experimental archaeology results. Additional information regarding the execution and details of the experiments can be found in the Supplementary Information.

### Rationale for choosing extraction methods

The point of departure for designing the experiments was to test the most feasible methods and materials known to be available to prehistoric hunter-gatherers. Seven methods were chosen based on previous archaeological and ethnographic research (Little et al. 2016; Domínguez-Solera 2018; Rainio and Tamboer 2018; Morrison et al. 2022; Langley et al. 2023) and the authors’ combined expertise of hunter-gatherer material culture, technology, zooarchaeology and taphonomic studies. Experiments (E) 1–7 included: cutting (E1), percussion (E2), scavenging/air-drying (E3), soaking (E4), direct heat/fire (E5), cooking—boiling (wet/ceramic) (E6), cooking—earth oven/steaming (E7). Hypotheses for the extraction methods were:

- E1: Lithic flakes/blades can be used to cut teeth from the mandible.
- E2: Crushing a mandible using cobbles and wooden implements percussively will loosen the teeth from their sockets.
- E3: Leaving a mandible outdoors for an extended period of time (several months) will cause the bone to dry out and the teeth can be extracted manually.
- E4: Soaking a mandible for several weeks will soften the bone and decompose the soft tissues, and the teeth can be extracted manually.
- E5: Exposing a mandible to direct heat from an open fire will make the bone dry and brittle, and the teeth can be extracted manually.
- E6: Simmering a mandible in a ceramic pot will cause the soft tissues to detach, and the teeth can be extracted manually.
- E7: Placing entire heads into an earth oven (cooking pit) will detach the soft tissues, and the teeth can be extracted manually.

While other methods could be tested, we argue that these seven are the most likely given the technologies available at this time. Since this area of research has faced limited attention, the literature on experimental extraction is sparse (for exceptions, see Rainio and Tamboer 2018; Rainio et al. 2021). Even though animal teeth are abundantly found in hunter-gatherer contexts, physical evidence for tooth extraction is relatively rarely presented in the literature. Cutting out animal teeth with flint tools is the most discussed method, notably in relation to red deer teeth pendants from the Palaeolithic and Mesolithic contexts (d´Errico and Vanharen 2002; d’Errico and Rigaud 2011; Rigaud 2013; Brinch Petersen 2015; Lass Jensen 2016; Tejero et al. 2021). Percussion as a suitable method for breaking bones has been mentioned and tested with various successes (Blasco et al. 2014; Barrett et al. 2020). Our air-drying experiment was designed to replicate scavenging—opportunistic collection is an obvious method for sourcing teeth, and the least intensive technologically. However, in a truly actualistic scavenging scenario, carcasses would be lying on the ground surface, exposed to moisture, insects, bacteria, and other decomposing agents (Andrews and Cook 1985), not being suspended/air-drying. In this respect, our experiment fails to replicate typical scavenging scenarios or other techniques to promote the decomposition process, such as intentional rotting by caching or burying animal body parts. Heating red deer crania through direct contact with hot embers has been shown to be successful in removing bone moisture, making the bone brittle and easier to manipulate with stone tools (Little et al. 2016). Therefore, it was decided to test whether this reduction in bone moisture by direct heat facilitates tooth extraction. Other methods of deconstructing skulls, including boiling and steaming during cooking activities related to food consumption, have been described in the ethnographic literature, specifically the breaking of antelope mandibles and skulls by the Ju/’hoansi (Domínguez-Solera 2018).

### Experimental program

#### Location, set up, ethics, materials and limitations

The experiments were conducted outdoors at the Īdeņa Experimental Centre, eastern Latvia, over a period of one year. The location was chosen because it enabled the sourcing of raw materials necessary for the experiments from licensed local hunters. It also allowed for longer-duration actualistic experiments involving animal remains, including months of soaking, which would not be possible in urban or university settings. In total, elements from eleven animals were used for the experiments: seven skulls or mandibles of Eurasian elk*,* two of wild boar*,* and two of roe deer (Tab. 1).

**Table 1:** Animal materials used for the experimental programme, including the individual animal number (AN), species, age, body part, condition and relevant experiment (E) number

Animal number (AN)	Species/Latin name	Estimated age	Anatomical part	Condition	Experiment (E) number
AN1	Wild boar/*Sus scrofa*	Adult male, > 2 years old	Head	Fresh, skinned, soft tissues remaining	E1, E2, E3
AN2	Wild boar/*Sus scrofa*	Adult male, > 2 years old	Head	Frozen, skinned, soft tissues remaining, snout with skin	E2, E7
AN3	Elk/*Alces alces*	Adult male, 3 years old	Head with antlers	Frozen, skinned, very little soft tissues remaining	E1, E5
AN4	Elk/*Alces alces*	Young male, < 1 year old	Mandible	Frozen, skin remaining on the mandible	E1, E5
AN5	Elk/*Alces alces*	Young male, < 1 year old	Mandible	Frozen, skin remaining on the mandible	E7
AN6	Elk/*Alces alces*	Young male, < 1 year old	Mandible (distal part with incisors)	Frozen, skinned, soft tissues remaining	E1
AN7	Roe deer/*Capreolus capreolus*	Adult male, > 2 years old	Head with antlers	Fresh, with skin remaining	E6, E7
AN8	Roe deer/*Capreolus capreolus*	Adult male, > 2 years old	Head with antlers	Fresh, with skin remaining	E7
AN9	Elk/*Alces alces*	Adult male, ca. 2.5 years old	Head with antlers	Fresh, skinned, soft tissues remaining	E4
AN10	Elk/*Alces alces*	Adult female, ca. 4 years old	Mandible	Fresh, skinned, soft tissues remaining	E2
AN11	Elk/*Alces alces*	Adult male, > 2 years old	Mandible	Fresh, skinned, soft tissues remaining	E2

The availability of raw materials was dependent on the local hunting yield, which affected the experimental programme, while the timing of conducting the experiments was also shaped by the Latvian hunting season. Sometimes the results were clear after the first run of the experiment (e.g., direct heat/fire, scavenging/air-drying, soaking, boiling, cooking pit), other times they needed repeating (cutting, percussion). Although the lack of repetition and standardisation is not considered ideal in most experimental programmes (Karr and Outram 2015), working with animal remains, often in different stages of decomposition, poses unique challenges, especially concerning ethics and health/safety. If the result of an experiment was sufficiently clear, repeating it was not deemed justified, avoiding the unnecessary use of animal materials. For the same reason, some raw materials were used for several experiments. The limited availability also meant that to ensure a large-enough sample, raw animal materials occasionally had to be frozen (6–8 weeks) until the experiments took place, while others were used in a fresh state (within a couple of hours of the animal’s death). Freezing bones can affect how they fracture and how resistant they are to both static and dynamic mechanical forces (Andrade et al. 2008; Karr and Outram 2015; Kaye et al. 2012; Tersigni 2007). By acknowledging these varying states of bone preservation, we aim to improve methodological standardization and thus contribute to the comparability and replicability of this study in the future (see Karr and Outram 2015 for similar discussion).

The first experiments took place in winter over 3 days with an average daily temperature of −2 °C and moderate snow cover. At this time, frozen heads and mandibles of elk and wild boar were available. Subsequent experiments were carried out during the summer season, again over 3 days, when weather conditions were milder, with an average temperature of 19 °C. At this time, we had access to fresh roe deer heads. The percussion and soaking experiments took place in the autumn when the elk hunting season had begun again and more fresh elk mandibles were available. The soaking experiment continued over a period of five months, with temperature fluctuating between 15 and −15 °C, while the percussion experiments took place over two days. No animals were killed for the purpose of this study: all remains were byproducts of animals consumed as part of everyday subsistence by local communities. The health, safety and ethics forms were processed through the Department of Archaeology, University of York.

## Teeth extraction methods

### E1 Cutting

#### Method, execution and results

**Fig. 7:**
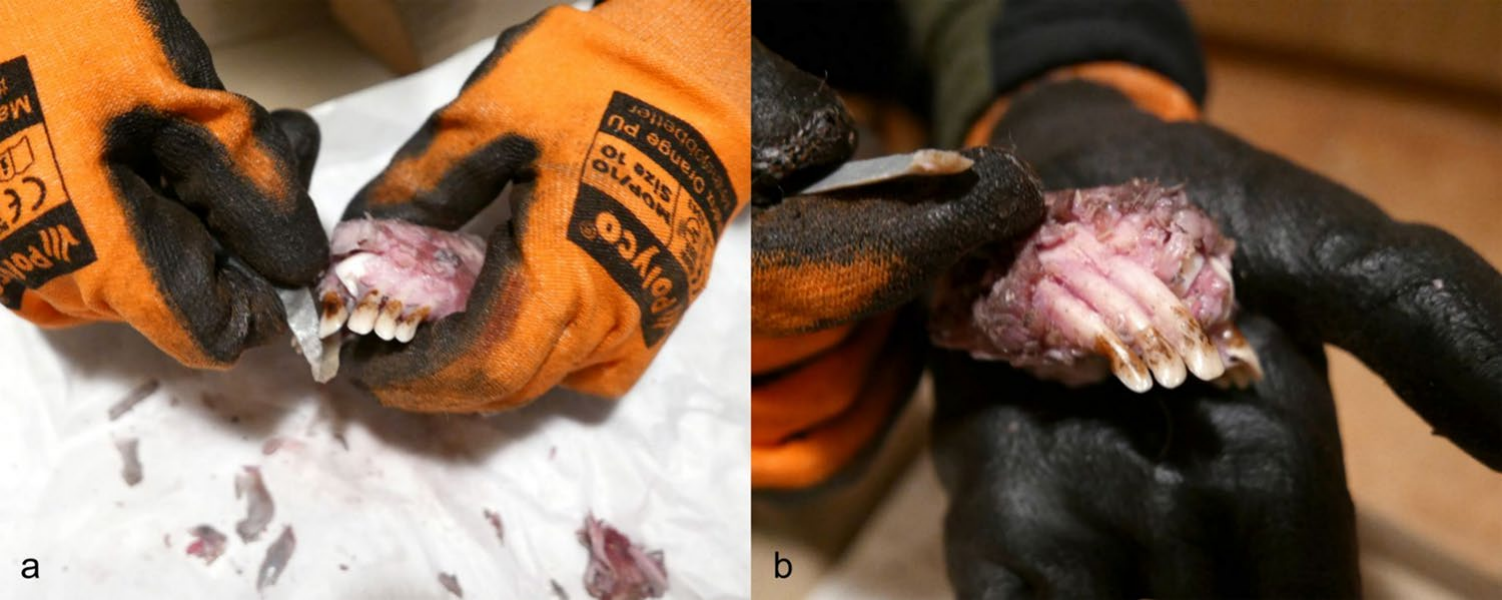
The cutting experiment (E1). **a** Unretouched flint blade was used to cut out incisors from the young elk calf mandible. **b** The extraction process was facilitated because the bone was soft, and the mandibular symphysis was still not completely fused. Photos by A. Macāne, illustration by K. Nordqvist

**Fig. 8:**
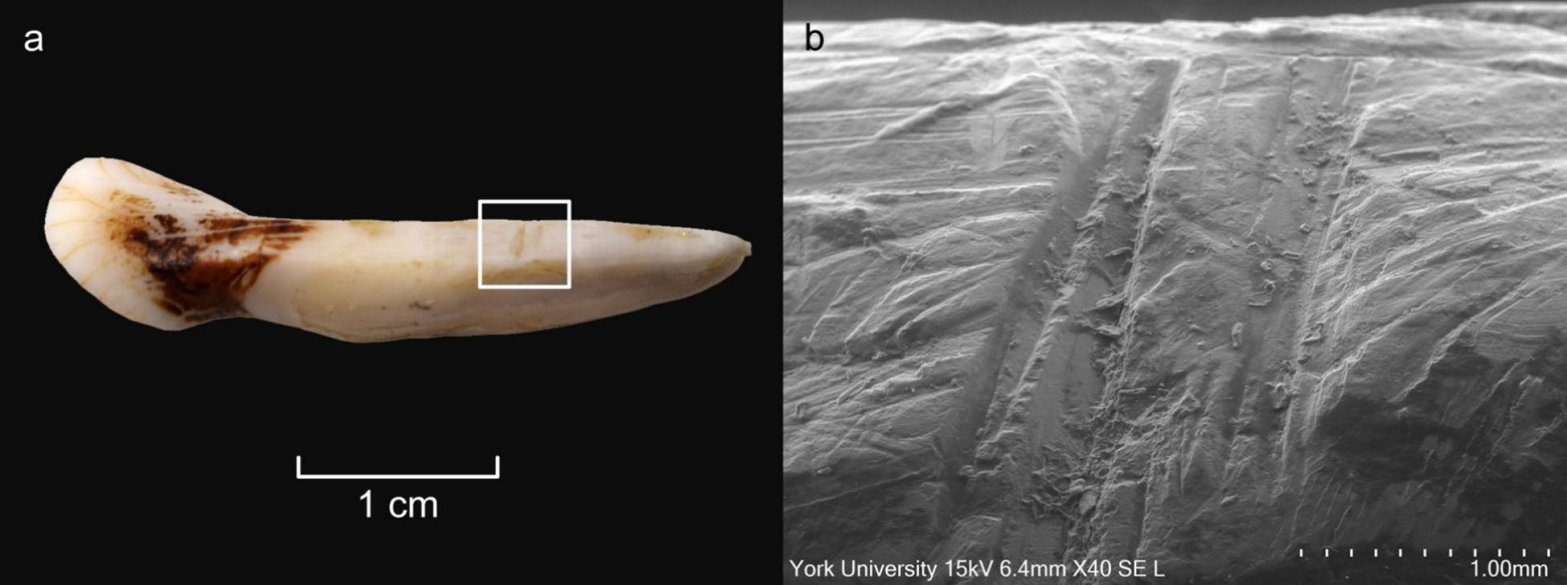
Results from the cutting experiment. **a** Cut marks visible to the naked eye on the crown and root of an elk deciduous incisor. **b** Cut marks on the root (marked with a white frame) magnified with a SEM. Photos by G. Perry, illustration by K. Nordqvist

Three elk mandibles (AN3, AN4 and AN6) and one wild boar (AN1) were used in the cutting experiment. Several unretouched flint flakes and blades were used to cut incisors from an adult elk (AN3) and a wild boar (AN1) mandible; however, it was not possible to extract the teeth (see Table S.I.1). The attempt to cut out teeth from a young elk (AN6) was more successful (Fig. 7a-b). During cutting, the mandible was divided in two halves (the mandibular symphysis was not yet fused), which facilitated easier access and the working angle. Nevertheless, the tooth extraction process was challenging—it took a total of 40 min to cut out the first incisor. Extraction is made difficult by the flint slipping on fresh bone and soft tissues. This method leaves traces both on the tooth and the mandible, with cut marks clearly visible even with the naked eye on the crown and root (Fig. 8a). Lack of experience (skills) may have affected the result. It is possible that with some practice the process could be sped up and leave less traces. In sum, the hypothesis is rejected for the Zvejnieki assemblage due to the lack of similar traces on crowns and roots as those created during our experiments (still, it cannot be excluded that cutmarks on the roots were ground away). It is, however, possible that the method is better suited for other animal species. Cutting has been documented elsewhere as a possible method for extracting red deer or reindeer teeth (see above and discussion below), though it seems unlikely that different sub-species of cervids would respond differently. Additionally, the age of an animal affects the bone morphology (Ioannidou 2003); our experiments indicate that the cutting method may be more suitable for younger animals with softer jaw bones.

### E2 Percussion

#### Method, execution and results

**Fig. 9:**
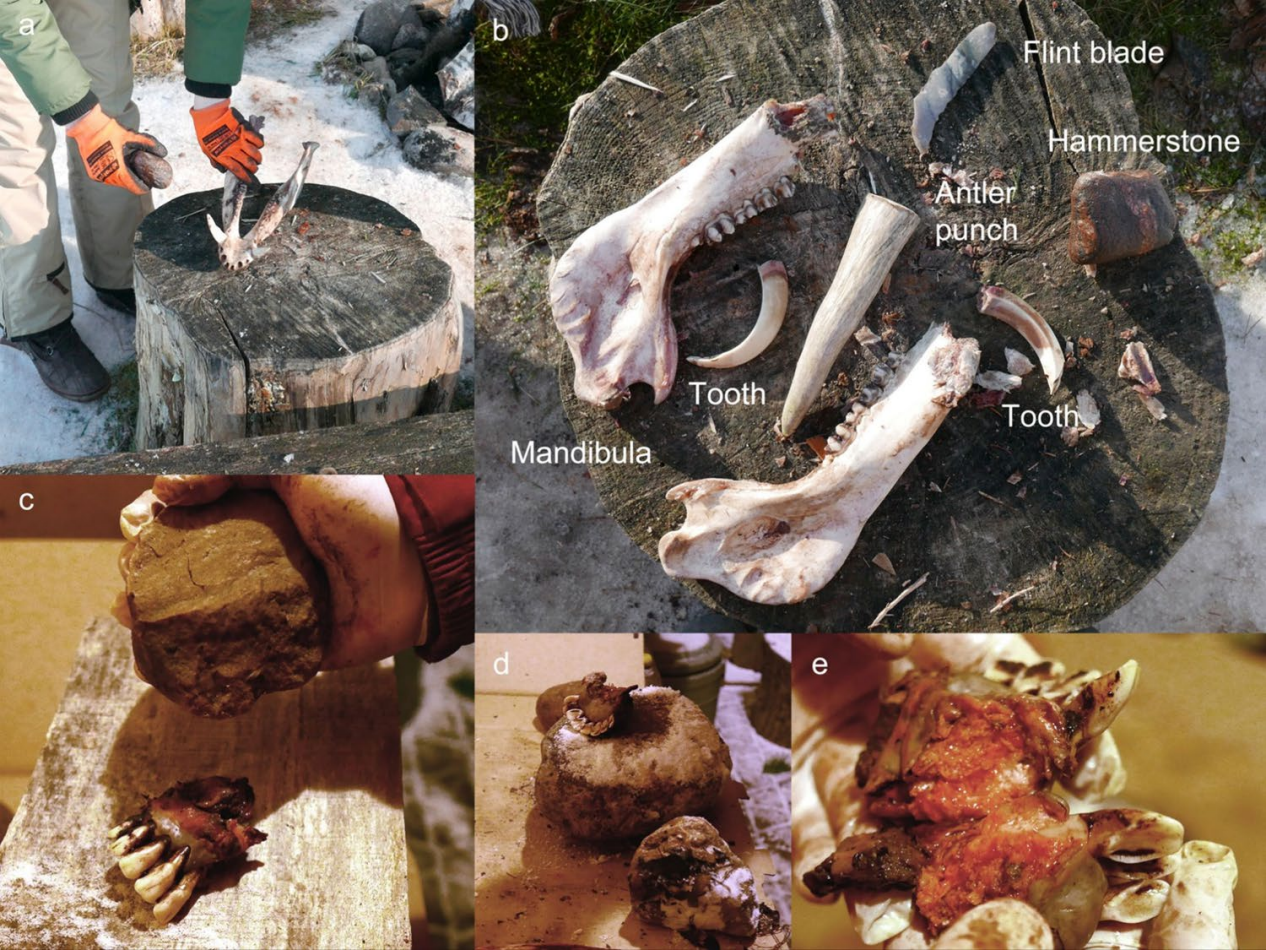
The percussion experiments (E2). **a** The extraction of wild boar canines using a rounded cobble. **b** Tools used to extract the wild boar canines. **c** A stone cobble and a wooden base were used for extraction of elk incisors. **d** A stone base was also tested during the percussion experiment. **e** Teeth often were damaged during the percussion experiments. Photos by A. Macāne and A. Little, illustration by K. Nordqvist

During the first experiment, the skull of a wild boar (AN1), previously used in the air-drying experiment (see E3), was hit repeatedly with a rounded cobble and piece of wood to extract the teeth from the skull. The percussion experiment was repeated three times (Table S.I.2.). Using a percussive action the wooden implement was used for 5 min, the cobble for 3 min. This method was also tested on the wild boar (AN2, Fig. 9a-b), however, the canines could not be removed. A tine was cut from an elk antler and used as an indirect punch to allow more precise percussion on the bone using a cobble as a hammerstone. This method proved to be successful since it directed the power to the desired point, crushing the bone but not damaging the teeth. The percussion experiment was repeated two more times with elk mandibles (AN10 and AN11), a rounded cobble and a stone with sharp edges, using both stone and wood as bases (Fig. 9c-d). First, the distal part of the mandible (mandibular symphysis) with the incisors was detached from the ramus mandibulae by striking it with a stone. Then, an angular stone was used to crush the mandible. After approximately 5 min, the mandible was crushed, but the teeth were still held in the mandible by soft tissue. Percussion was continued until the teeth were extracted from the alveoli. The elk incisors were extracted, confirming the hypothesis; however, some incisors were partly missing the root or crown and had soft tissues (periodontal ligaments; see Fig. 6) still attached (Fig. 9e).

**Fig. 10:**
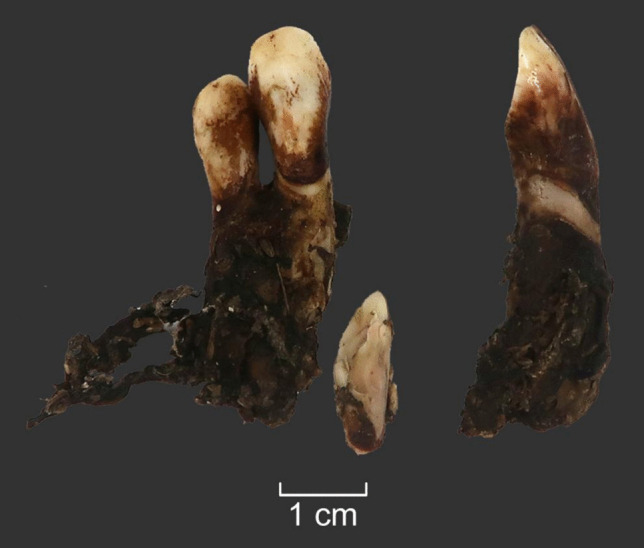
Elk incisors after the percussion experiment. The soft tissues are still attached to the root and in some cases the root end was broken because of this method. Photos by A. Macāne, illustration by K. Nordqvist

This method is feasible using stones with sharp edges and a reasonable degree of strength, accepting the latter is subjective. While experience and skill may improve the chances of extraction, the method is high risk because it comes with a strong possibility of teeth being damaged during the process (Fig. 10). It also involves an extra stage of cleaning the periodontal ligaments that remain attached to the tooth once extracted. Further boiling, grinding or scraping is required to remove them, with grinding and scraping likely to leave traces on the tooth surface. Certainly, the rounded hammerstone was less effective than a stone with sharper edges: a sharp edge enabled better control over the point of impact and penetrated the bone more easily. Wood substrate required more percussive power because it is softer than stone.

### E3 Scavenging/air-drying

#### Method, execution and results

**Fig. 11:**
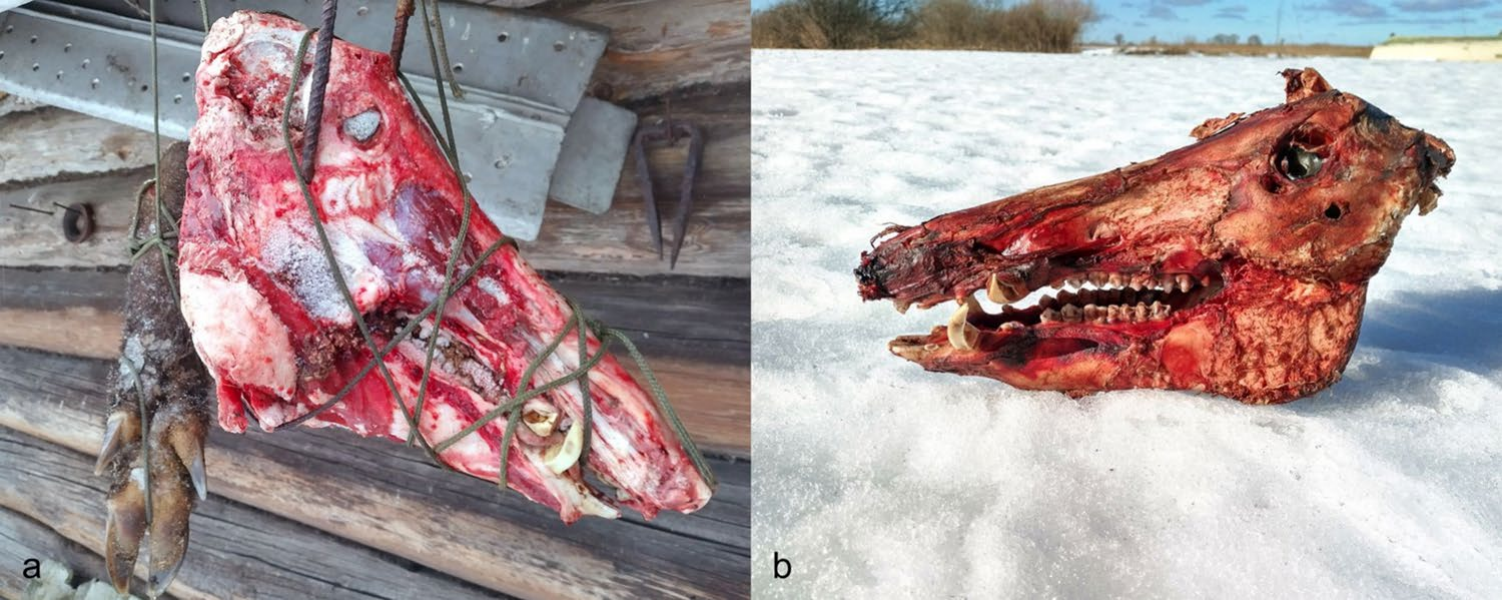
The air-drying experiment (E3). **a** A wild boar skull was hung in the open air for two months, exposed to different elements and temperatures. **b** After two months, the teeth were still tightly sitting in the jaw and could not be moved. Photos by A. Macāne, illustration by K. Nordqvist

To test the possibility of collecting animal teeth by air-drying, we hung a wild boar head (AN1, later used in E1 and E2) outdoors for two months, exposing it to sun, rain, snow and temperature shifts from −20 to 8 degrees Celsius (Fig. 11). The objective was to decompose the soft tissues and dry out the bone so that the teeth would loosen and become easy to remove. The head had to be hung, otherwise it would likely have been scavenged by other animals, which underscores the low likelihood that scavenging was the primary method for animal teeth sourcing at Zvejnieki. After two months, an attempt was made to pull out the teeth from the air-dried wild boar mandible — this was unsuccessful.

Hanging the wild boar head in the winter season may have impacted our ability to extract teeth. Flesh and other soft tissues air-dried, which apparently led to the teeth becoming more firmly connected to the alveolar yokes. It is possible that a warmer time of the year would be more successful, e.g., summer, with more insects, higher temperatures and rain. Alternatively, hanging and exposing to the elements for a much longer time to decompose all the soft tissues and dry the bone more might facilitate the extraction of the teeth from the alveoli. As mentioned, contact with the ground (or lying in water) would expose the head to decomposing agents, speeding up the process (Andrews and Cook 1985; King and Birch 2015). However, the un-monitored drying and movement of a complete crania or mandible, either by taphonomic processes or scavenging animal interference, could lead to incisors falling out and being lost. Incisors and vestigial canines of red deer are lost quickly after the death of the animal (d’Errico and Rigaud 2011; Tejero et al. 2021), thus making the collection of teeth from older carcasses less likely than from recently hunted kills. In addition, teeth from carcasses that have been lying around for extended periods of time might display traces of surface weathering, though individual characteristics of each tooth, including stage of eruption, wear, ratio of enamel to dentine and overall morphology, can differentially affect weathering condition (Behrensmeyer 1978: 153).

### E4 Soaking

#### Method, execution and results

**Fig. 12:**
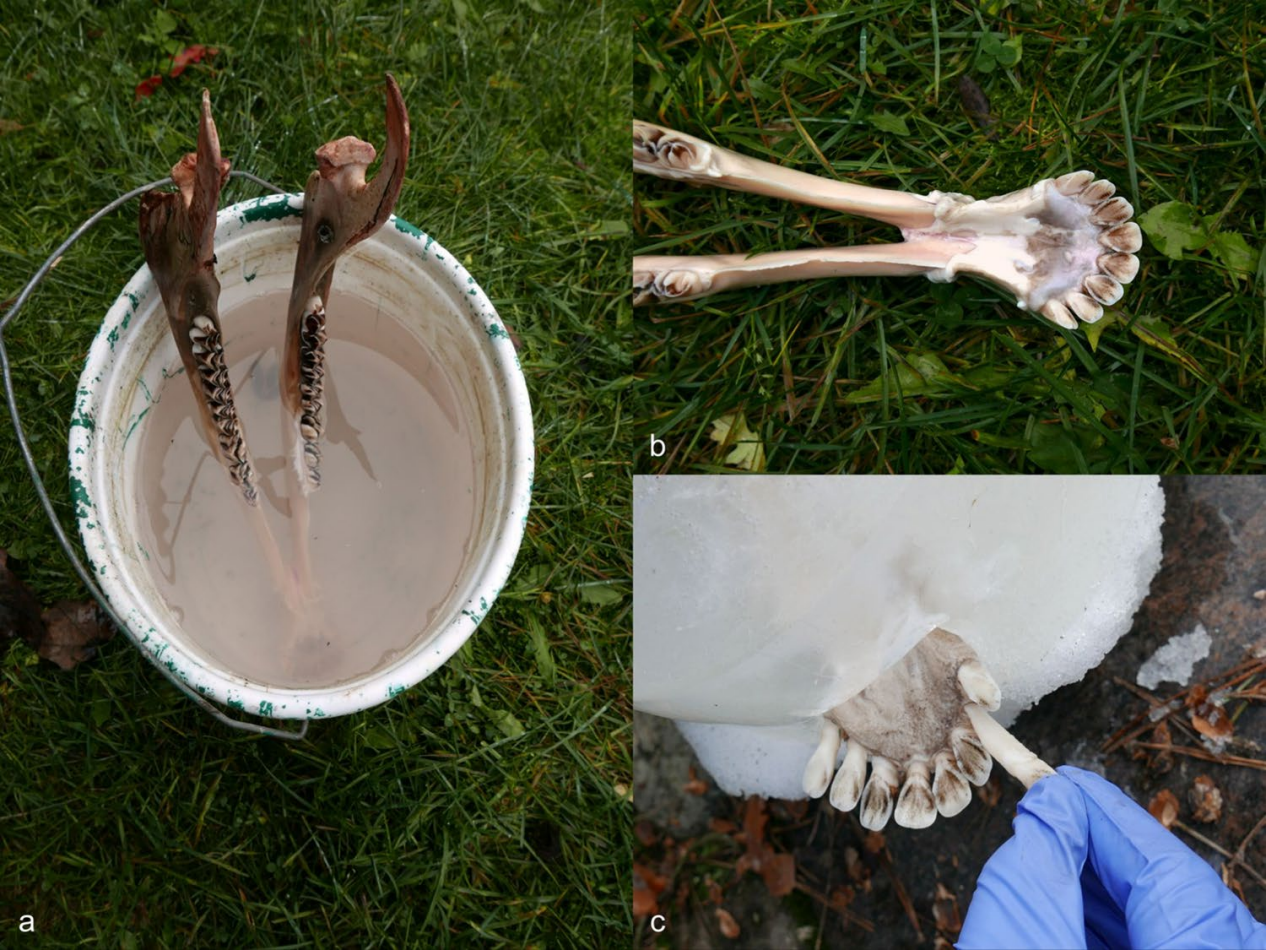
The soaking experiment (E4). **a** An elk mandible was soaked in a bucket with water for 5 weeks. **b** Extraction of incisors was attempted regularly but was unsuccessful. **c** The water froze during the winter and after 5 months, the teeth were easily pulled out from the jaw; some teeth still had periodontal ligaments attached (see Fig. 6). Photos by A. Macāne, illustration by K. Nordqvist

**Fig. 13:**
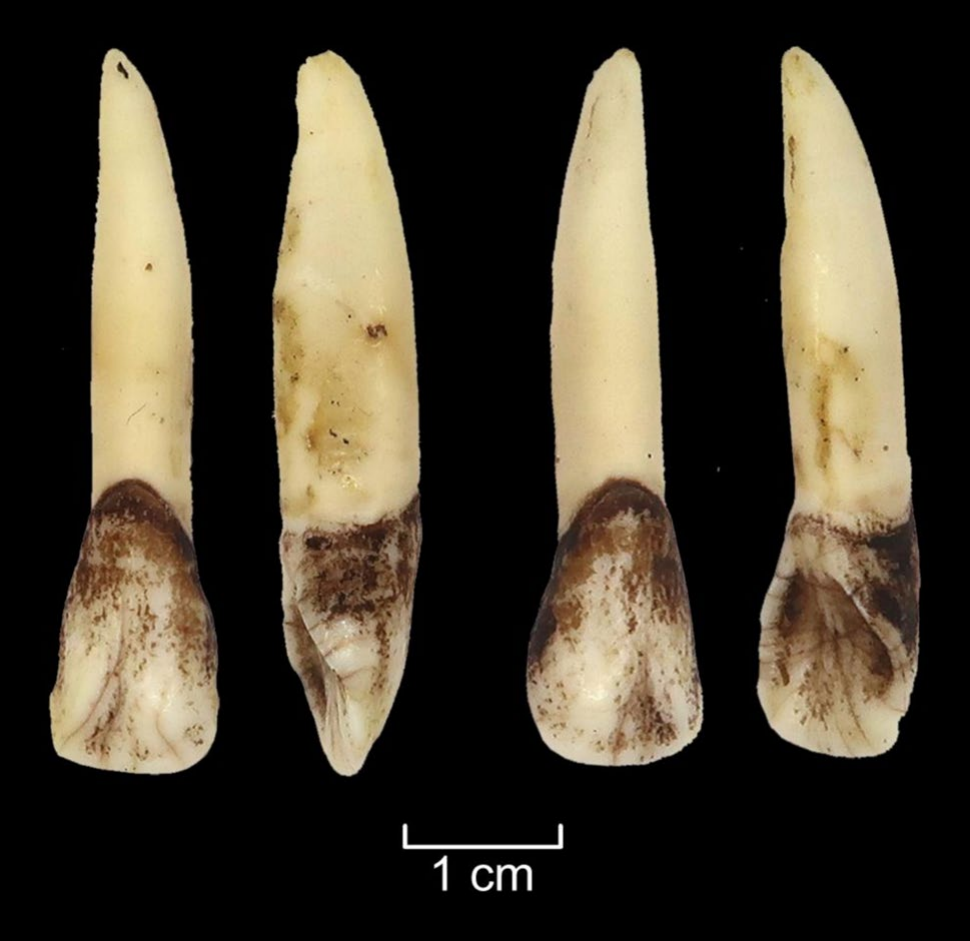
Elk first incisors from the left and right side from the soaking experiment after 5 months and freezing were virtually clean of ligaments and other soft tissues and did not display any signs of damage. Photos by A. Macāne, illustration by K. Nordqvist

An elk mandible (AN9) was used for the soaking experiment (Table S.I.3.). The skinned mandible was placed in a bucket, filled with local pond water (Fig. 12a). The distal end of the mandible (with incisors) was submerged, the proximal end was not. The bucket was then placed in the open air where insects and bacteria could reach it, and it was protected from wild and domestic animals. The experiment took place during autumn when temperatures outside varied from 15 to 0 degrees Celsius. Over five weeks, the attempt to pull out the incisors was repeated four times (at intervals of 7–12-days), without success (Fig. 12b). The soft tissues rotted away, however the gingival cuff held the teeth firmly in place, even if they were moving slightly. The experiment was interrupted by severe frost (up to −15 degrees Celsius), requiring the mandible to be left in frozen water over the winter. The next attempt to extract the teeth occurred after five months. By this time, the ice had begun to melt, and it was possible to remove the mandible from the bucket. The teeth were then pulled out from the alveoli without any damage (Fig. 13) but occasionally with the periodontal ligament still attached (Fig. 12c).

Soaking an elk mandible in water proved to be a time-consuming experiment during the colder part of the year. Under warmer conditions with increased bacterial and insect activity, the process might have been completed more quickly. Assessing the impact of frost on the tooth extraction process is challenging, as it is unclear whether it facilitated or delayed the process. Additionally, using a vessel for soaking may be preferable; theoretically, the mandible could also be left in a natural water body. However, this approach would risk exposure to scavengers. On the other hand, this might also increase exposure to insects and bacteria, which could be beneficial for the process.

In sum, we can confirm the hypothesis E4; however, the viability of this method is questioned, given the need for secure storage conditions from scavengers. This method requires follow-on cleaning since soft tissues adhered to some of the roots. Despite these limitations, soaking the mandible (including placing it in an open water body) may have been advantageous for preserving the different raw materials (teeth, mandible) and meat (e.g., Speth 2017) for later use. Of course, depositing decaying animal remains in water supplies would not be ideal for sanitation.

### E5 Direct heat/fire

#### Method, execution and results

**Fig. 14:**
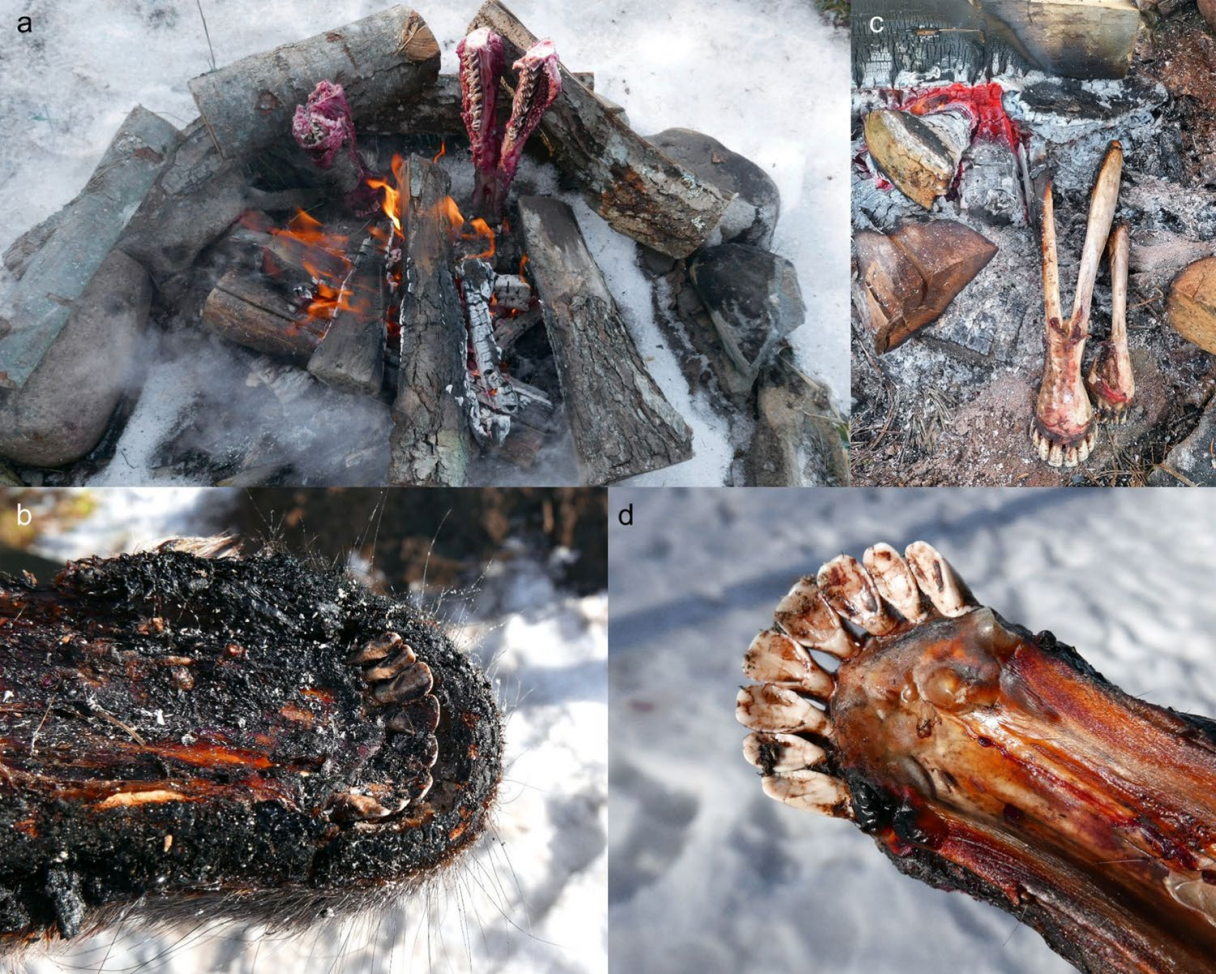
The direct heat experiment (E5). **a** Adult and young elk mandibles (AN3 and AN4) were placed next to open fire; **b** Within 30 min, the skin and soft tissues were burnt; **c** After two hours, AN3 and AN4 were removed from the fire and cleaned. After an unsuccessful extraction attempt, the mandibles were placed back into the embers, with incisors facing away from the fire; **d** The gingival cuff effectively gelatinized, adhering the teeth to the alveoli. Photos by A. Macāne, illustration by K. Nordqvist

**Fig. 15:**
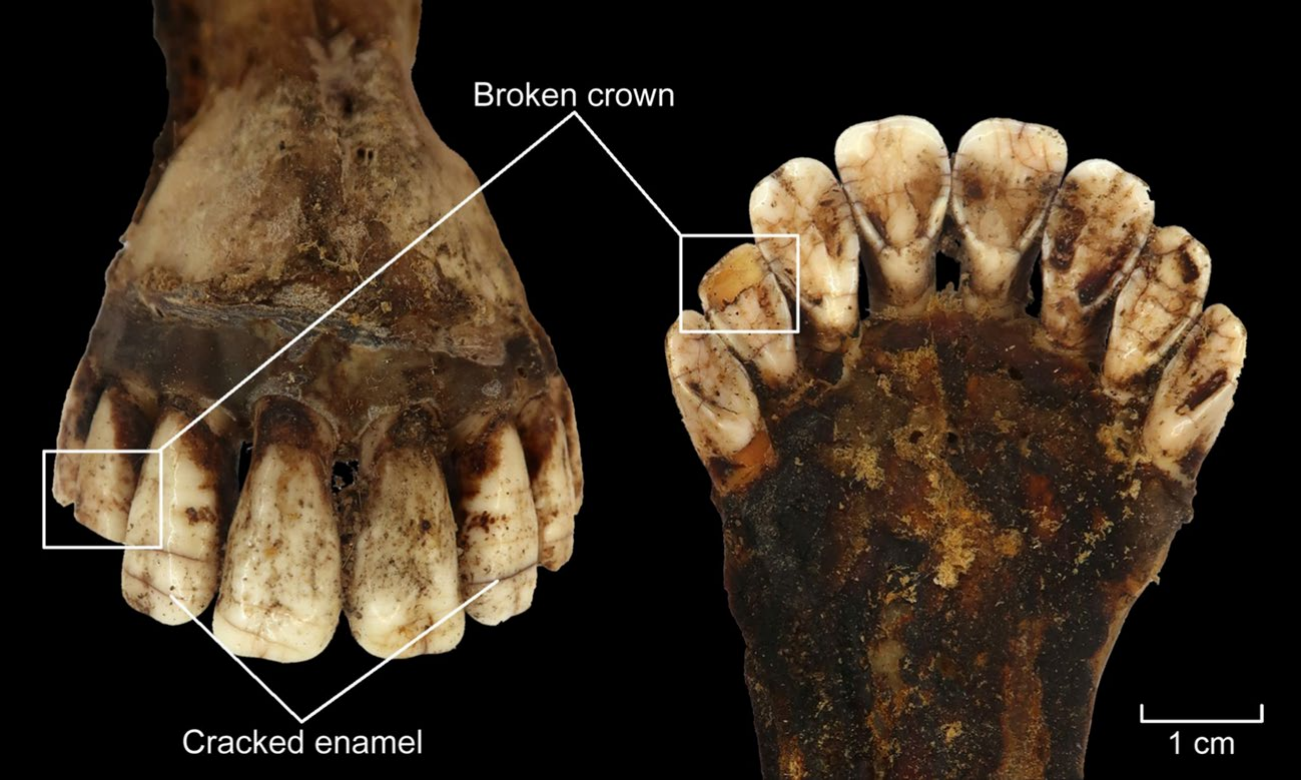
Impact of fire, including horizontal lines on enamel, was visible on the incisors and mandible of the adult elk after the direct heating experiment. The gingival cuff gelatinized into glue, keeping the incisors firmly in the alveoli and causing the crown of the third left incisor to crack during an attempt to extract it. Photos by A. Macāne, illustration by K. Nordqvist

Two elk mandibles were used in this experiment: AN3 and AN4 (Fig. 14a). The mandibles were placed in an open hearth, sitting in the embers, approximately 20 cm away from the fire (for more details see Table S.I.4.). When the skin and soft tissues began to burn, the mandibles were moved further away from the flames. After 30 min, an attempt was made to extract teeth, but the incisors (and all other teeth) were not moving. Extraction was tried again after two more hours by the fire but was again unsuccessful. In fact, the fourth incisor of AN4 broke during this attempt. Burnt skin and soft tissues (Fig. 14b) were cleaned from AN3 and AN4 before putting them back into the embers, with the incisors facing away from the fire (Fig. 14c). After three hours, a third attempt of extraction was unsuccessful and resulted in a partly broken crown of the third left incisor of AN3, with horizontal lines observed on the crowns due to the heat (Fig. 15).

Direct heat caused the tooth enamel to become brittle (Fig. 15) and the teeth became glued in the alveoli as the gingival cuff heated up, gelatinizing, creating a type of collagen glue (Bleicher et al. 2015) (Fig. 14d, Fig. 15). Problematically, this method damaged the teeth even before we could attempt to pull them out of the mandible; it also compromised the usability of the bone for other purposes. There are no similarities with the Zvejnieki assemblage, as no evidence of burning was observed on the archaeological tooth pendants. On this basis, the hypothesis is rejected.

### E6 Cooking—boiling (wet/ceramic)

#### Method, execution and results

**Fig. 16:**
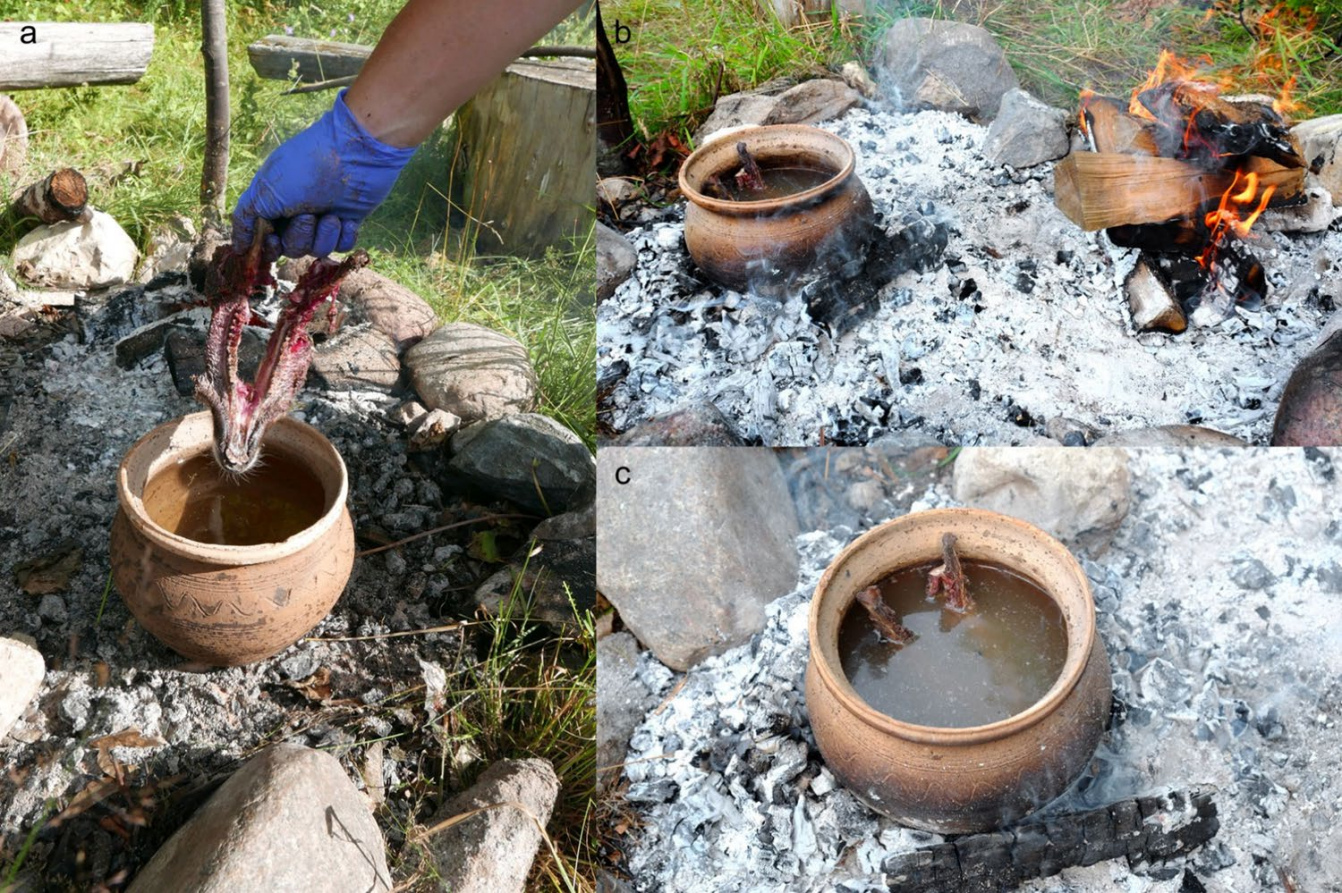
The boiling experiment (E6). **a** A roe deer mandible was cooked in a replica ceramic vessel. **b** The heat around the pot was gradually increased. **c** After 10 h of boiling, the teeth were successfully removed undamaged. Photos by A. Macāne, illustration by K. Nordqvist

**Fig. 17:**
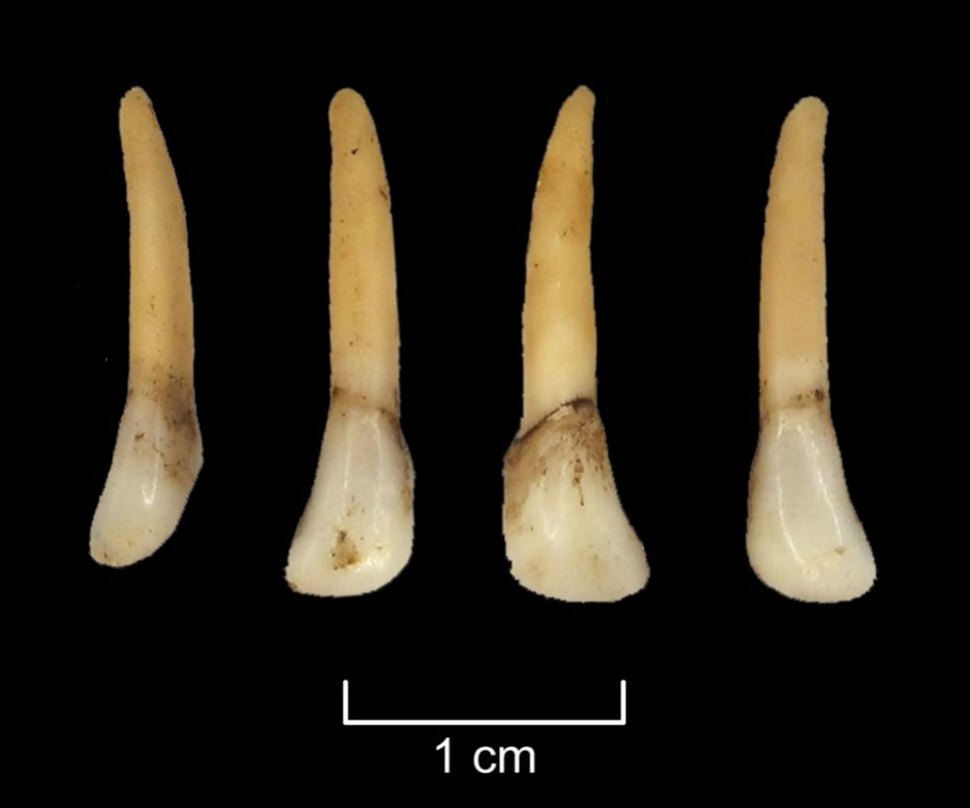
The roe deer incisors extracted in the boiling experiment (E6) came out clean and without any visible damage. Photos by A. Macāne, illustration by K. Nordqvist

Many of the animal teeth from the Zvejnieki cemetery come from a time when ceramic vessels were in use (pottery was introduced in the late 6th millennium cal BC; Meadows et al. 2018; Zagorska 2006). Therefore, an attempt was made to boil a roe deer mandible (AN7) in a ceramic vessel (Fig. 16). A replica of a prehistoric hand-made pottery vessel was used. The pot, containing water, was slowly warmed up near a fire to avoid breakage from thermal shock. Hot embers were then placed around it and regularly added to maintain the boiling temperature (see Table S.I.5.). Roe deer mandible (AN7) was placed in the pot and slowly cooked for more than 10 h to test whether the teeth could be loosened/extracted at different stages. Even though boiling was a long and fuel-intensive process, this method was efficient and did not damage the teeth (Fig. 17). They could be easily extracted while the mandible was still warm; however, like the earth oven experiment (E7, see below), the teeth quickly became “glued” back to the bone once it started to cool down. Boiling with a pottery vessel took a lot of time, partly due to concerns around rapid heating breaking the pot—cooking time could be potentially reduced by adding fuel with greater frequency.

### E7 Cooking—steaming/earth oven

#### Method, execution and results

**Fig. 18:**
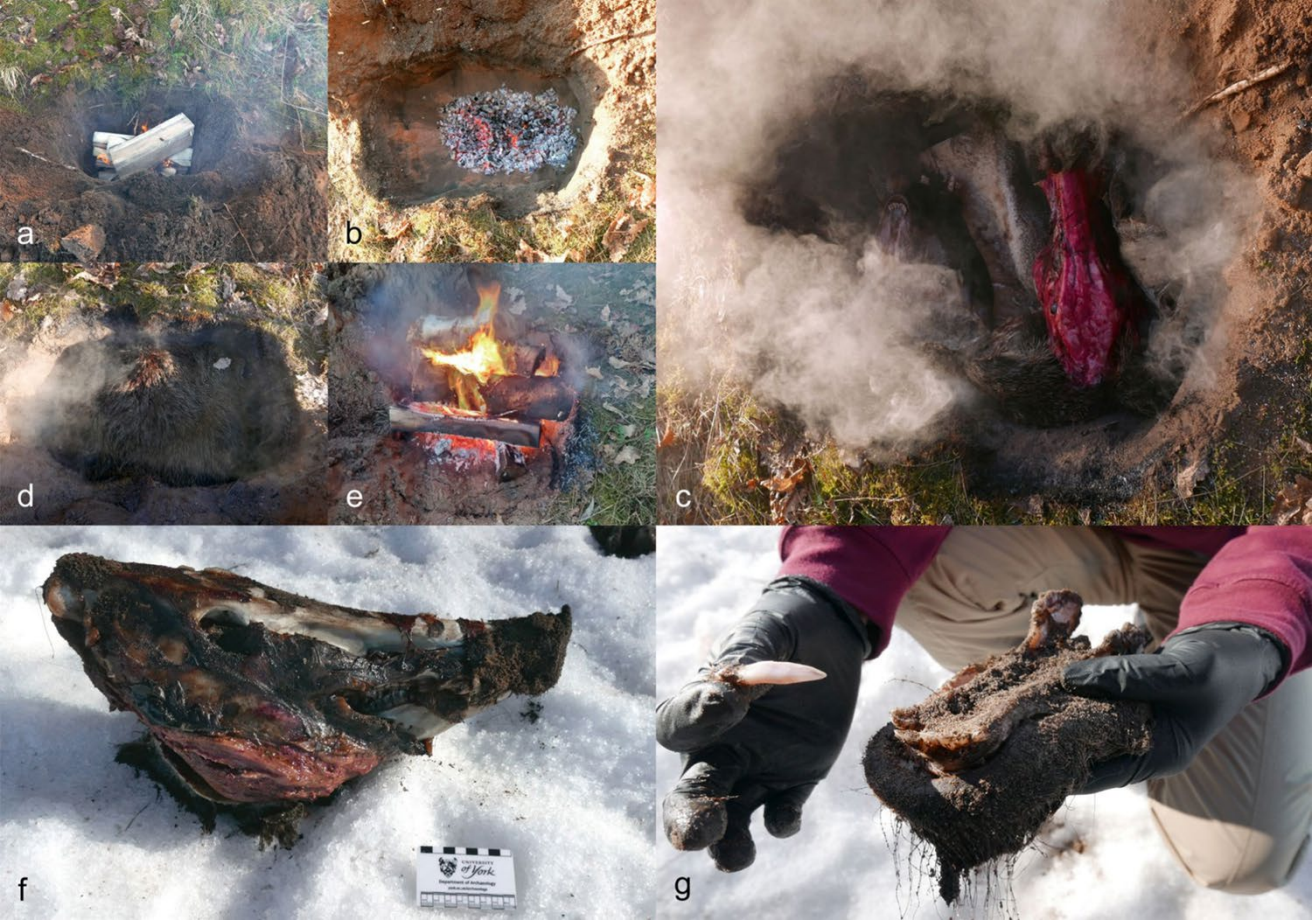
The cooking pit winter experiment (E7). **a** First, a fire was started in a pit lined with stones. **b** When the charcoal was ready, **c** half a wild boar skin was placed over it, with a wild boar head (AN2) and an elk mandible (AN5) on top. **d** The remaining wild boar skin was then placed over the head and mandible and snow was added to enhance the steaming process. **e** A thin layer of sand was placed on the wild boar skin and a new fire was lit on it. **f** After opening the pit, the wild boar head was thoroughly cooked. **g** The incisors were easily removed from the alveoli; no visible traces were left on the teeth or mandibles. Photos by A. Macāne and A. Little, illustration by K. Nordqvist

An earth oven (a cooking pit) was used to steam-cook various animal heads for 13–17.5 h (for more details, see Table S.I.6. and Table S.I.7.). This experiment was run twice due to a thermocouple failure during the first attempt. The first (winter) experiment included an adult wild boar (AN2) and an elk calf (AN5) (Fig. 18); in the second (summer) run, a roe deer head with antlers (AN8) and a skull without the mandible (AN7) were used.

A pit measuring 85 cm by 50 cm and 60 cm deep was dug in frozen sandy soil. Small stones/cobbles (approx. 10–20 cm in diameter) were used to line the bottom of the pit, followed by a layer of broadleaf wood for fuel. A fire was started above the stone layer and burned for four hours (Fig. 18a-b). Half a wild boar skin was then placed over the hot embers for insulation. A wild boar head (AN2) — skinned but with the snout, meat, and tongue intact—and AN5, the mandible of an elk calf (with skin and lip attached), were placed on the boar skin, with some snow added for moisture (Fig. 18c). The other half of the boar skin was used to cover the heads (Fig. 18d). A thin, 5–10 cm layer of sand was applied on top of the skin and a new fire (using the same type of wood as fuel) was started above the heads (Fig. 18e). After burning for two and a half hours, the resulting embers were covered with a layer of turf (7–10 cm) and a thin layer of sand. The oven was opened after 17.5 h. At this point, the wild boar head was thoroughly cooked; the insulation with wild boar skin and snow had facilitated the steaming/cooking process (Fig. 18f). Tooth extraction was carried out within 10–15 min after the heads were lifted from the oven and allowed to cool down slightly. At this point, the incisors were easily extracted from the alveoli (Fig. 18g). Soon afterwards, within 10 min, we attempted to remove the canines, but the teeth were sitting tightly in the mandible and their extraction required the use of additional tools (see E2 above).

**Fig. 19:**
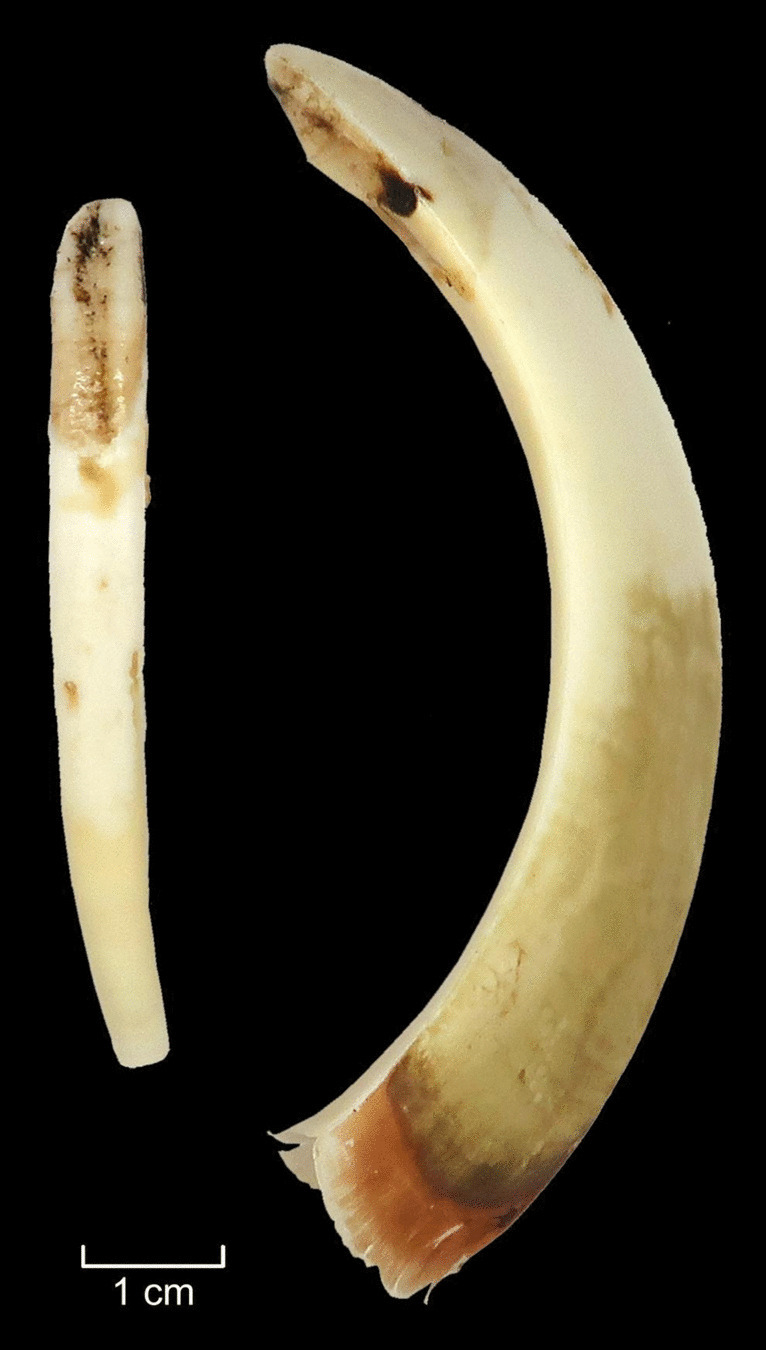
Wild boar incisor and canine extracted in the cooking pit experiment (E7). Photos by A. Macāne, illustration by K. Nordqvist

For the second attempt, carried out in the summer, the same cooking pit was reopened and the same cobbles were used. A fire was made once again on the cobbles and after the charcoal was ready, a roe deer skin was used as insulation. Two roe deer heads (AN7 and AN8) were placed on the skin and half a litre of water was poured over them. A thick layer of ferns (*Pteridium aquilinum*) was then placed on top. The soil excavated from the oven pit was backfilled, covering the layer of ferns, and another fire was lit. After three hours of burning, the charcoal was covered with turf. The oven was left overnight and opened after 13 h. The mandible (AN8) was detached from the head, resulting in the division of the unfused mandible into two halves, which further facilitated the extraction process. The incisors (AN8) were easily pulled out shortly after the head was taken out from the pit. The skin and soft tissues were also easily removed from the remaining cranial bones. The meat underneath was thoroughly cooked. The heads, along with the skin, protected the meat from dirt and sped up the cooking process. In sum, this method enabled teeth to be removed with ease (Fig. 18g, Fig. 19), with the condition of speed being a significant factor, confirming the hypothesis.

## Experiments (E1–7) results summarised

Our experiments indicate that the three methods of extraction—E4 (soaking), E6 (boiling using a ceramic vessel) and E7 (steaming in an earth oven)—were successful. This means that the hypothesis of manual teeth extraction was achieved without damage being caused to the teeth during the process (see Table 2). In addition, two more methods E1 (cutting) and E2 (percussion), were viable, but teeth became damaged during the extraction. Two other tested methods (E3 and E5) did not provide successful results and are therefore considered less viable; however, scavenging from ground surfaces, cached material or longer air-drying, at different temperatures, cannot be fully excluded.

**Table 2:** Summary of the experimental results and comparison with the archaeological evidence from the Zvejnieki cemetery tooth pendant collection

Experiment number	Method	Hypothesis confirmed or rejected?	Similarity to teeth in the Zvejnieki collection	Diagnostic wear traces
E1	Cutting	Confirmed	Low. This method may have been used, with cutting traces subsequently eliminated by grinding	Cutmarks
E2	Percussion	Confirmed	Low. This method may have been used, however, the remaining soft tissues would have to be cleaned away, e.g., by grinding or boiling	Crushed/fragmented bones, mandibles, and skulls
E3	Air-drying	Rejected	Low. This method may have been used opportunistically; however, it provides limited chances to retrieve complete sets of teeth from the same animal	N/A
E4	Soaking	Confirmed	High. Similar lack of technological traces observed	No macroscopically visible traces of extraction; however, sometimes soft tissues need to be cleaned away, which might leave grinding or scraping traces
E5	Direct heat/fire	Rejected	Low. No similar traces observed	Fire-cracked teeth and bones
E6	Cooking/boiling (pot)	Confirmed	High. Similar lack of technological traces observed	No macroscopically visible traces of extraction
E7	Cooking/steaming (earth oven)	Confirmed	High. Similar lack of technological traces observed	No macroscopically visible traces of extraction

## Discussion

### Early stages in the teeth pendant *chaîne opératoire* and zooarchaeological signatures of animal teeth pendant manufacture

Reports on prehistoric teeth extraction techniques are rare. The most frequently published references of extraction methods and associated physical traces come from red deer teeth. It has been suggested that these were cut out as a set, which resulted in distinctive cut marks on the teeth. This has been proposed for the pendants from Aven des Iboussieres, France (d´Errico and Vanharen 2002), Große Ofnet, Germany (Rigaud 2013), and Vedbæk, Denmark (Brinch Petersen 2015; Lass Jensen 2016). Other cases of red deer teeth displaying cut marks, regarded as evidence of removal by the cutting method, include the Gravettian site of Sire, France (d’Errico and Rigaud 2011), and Dzudzuana and Satsurblia (Georgia) (Tejero et al. 2021).

Cutting of the tooth and then snapping it out of the jaw has also been reported, including horse canines at the site of Oelknitz (Germany) (Brasser 2012) and reindeer incisors at the site of Gönnersdorf (Germany) (Poplin 1972). Human teeth have similarly been noted to display cut marks, indicating that in some cases they may have been intentionally removed from fleshed cadavers (Notterpek 2022; White 2007) and suggesting parallel treatment of human and animal body parts (Birouste et al. 2015). The ethnographic record from the Canadian Arctic shows the use of whole sets of red deer teeth in decorations, such as griddle belts. In these cases, the front teeth were extracted from mandibles by cutting them out, but including and retaining the gingival cuff (Brinch Petersen 2015; Poplin 1983; Rigaud et al. 2019; Stephan 2015). The use of complete sets of animal teeth has been observed at Zvejnieki as well as in other prehistoric contexts in Europe (Macāne 2022; Petrović et al. 2024); it is possible that some of them also retained the gingival cuff. However, the Zvejnieki tooth pendant assemblage does not display oblique fractures at the tooth roots, which have been associated with the removal of the entire sets of teeth still retained in the gingival cuff, as has been observed elsewhere (Poplin 1983; Rigaud et al. 2019).

A key limitation of many previous studies is that they work on the premise that 1) cutting a tooth from a mandible is an easy/simple undertaking and that 2) the cut marks visible on the teeth resulted from the extraction and the manufacturing process. Regarding point 1, our experiments have showed that it is not easy to cut a mandible, whether fresh, air-dried, or heated, and we rejected this method, at least for the adult individuals and species we tested. Concerning point 2, it is only partially acknowledged that while cut marks on the teeth might well be related to extraction for the purpose of personal ornament production, they might also be incidentally produced during butchery, such as the cutting off the tongue for dietary purposes (Binford 1981; Poplin 1983; Rigaud 2013; Gaudzinski-Windheuser et al. 2023). Differences have been distinguished between the cutmarks on the buccal and lingual sides. The former are linked to the cutting of the gingival ligament (Rigaud 2013), and the latter to the tongue extraction (Binford 1981; Rigaud 2013). It is also possible that cleaning of adhering soft tissues may leave incidental marks that are not directly caused by the extraction process. Taken together, it is not straightforward to conclude that cutmarks correspond to tooth extraction in all cases. Experiments exploring the traces left by the release of soft tissues and the cleaning of teeth would be valuable.

The results from our experiments suggest the need to explore these practices more dynamically. Many of the extraction methods we employed created diagnostic traces (Table 2, SI) on the teeth and skulls, while in other cases their recognition could be difficult. Cutting with flint tools leaves traces on both the roots and crowns of the teeth, as well as on the mandibles. Impact/crushing traces from hammering with cobbles or wood should also be evident. Percussion experiments produced both fractured mandibles and broken teeth. Direct heating of the teeth increased their brittleness and left distinctive traces on their surfaces (see Fig. 15). While burnt bones (and teeth) are relatively easy to identify in the archaeological record, efforts have been made to distinguish bones cooked by steaming or boiling methods in archaeological assemblages using Transmission Electron Microscopy (TEM) (Koon et al. 2010). However, the identification of teeth that were cooked/steamed *specifically* in an earth oven is not possible. Furthermore, taphonomic conditions and post-excavation treatments (such as conservation substances, glues, and wax) affect the condition of the tooth pendants, complicating further analysis. Zooarchaeologists working on prehistoric faunal assemblages, whether from northern Europe or beyond, may encounter these diagnostic traces, depending on the method of extraction used. Reporting such traces may advance understanding of tooth extraction practices, as may further experiments to augment sample size and confirm results.

Despite the number of questions raised by our experimental work, we hope this study encourages a deeper consideration of what we argue has historically been a disconnect between the faunal record, zooarchaeological analysis, material culture studies, and the role of taphonomy in tooth pendant production. Further exploration of the faunal record for damage patterns on skulls could enhance understanding of tooth extraction processes and help distinguish between evidence of butchering and carcass processing, in addition to varying attitudes toward different animal species.

### Zvejnieki teeth pendants—extraction methods and broader social implications

By evaluating the results of our experiments and comparing them to the material found at Zvejnieki, we can identify several likely methods of tooth extraction. The least successful method in our experiments was the use of direct heat or fire. When heated, the teeth become brittle, leaving traces on the crowns that are not visible on the archaeological tooth pendants or the faunal assemblages from the settlement or cemetery. In fact, the method was demonstrated as unviable, damaging the teeth and glueing them in the alveoli. Furthermore, teeth—being very hard and sharp—are a useful raw material utilised for various purposes, including as tools. Damaging them therefore seems unlikely (see Roth 1900: 36–37 cited in Morrison et al. 2022).

We also reject the air-drying hypothesis as a primary method for teeth sourcing at Zvejnieki, although occasional scavenging cannot be ruled out. Dead animal bodies, whether injured, attacked, or died of natural causes, could have been encountered in the landscape. If the soft tissues had sufficiently decomposed, it may have been possible to extract the teeth. Although this method of sourcing animal teeth for pendants is possible, the integrity of complete sets of teeth is compromised by taphonomic factors, including predators, leading to a more opportunistic selection process. For these reasons, it is hard to conceive that the many hundreds of teeth from Zvejnieki, some of which are part of complete sets, were primarily sourced in this way.

Cutting and percussion are also viable methods, despite the potential damage they may cause to the teeth. With practice, one can improve the skill to deliver a more controlled impact using a punch (i.e., a tine and hammerstone), thus reducing damage to the teeth. If these methods were employed, identifiable traces on teeth and mandibles might be observable in the faunal assemblage of the settlement. The periodontal ligaments remained still attached (Fig. 10) during the percussion experiments and would have involved further cleaning to remove them, either by boiling, grinding or scraping, with scraping and grinding likely to leave traces on the tooth surface (see Rainio et al. 2021). The cut marks caused during the extraction experiment are dissimilar to the grooves documented on tooth roots in the Zvejnieki burials; the former are less uniform and less deep. It is, however, possible that this method is better suited for other animal species, since cutting is documented elsewhere as a probable method for extracting red deer or reindeer teeth. Our experiments also showed that the age of the animal affected the extraction success. Mandibles of younger individuals with softer bone morphology (see Ioannidou 2003) were more easily cut.

While soaking (E4) and wet cooking (E6) are among the most successful extraction methods, they have certain limitations. Soaking requires a secure watery location where the mandibles can be protected from scavengers and other disturbances. Additionally, the site must be chosen carefully to prevent contamination of water sources with rotting animal remains. Although non-ceramic vessels made from wood or other materials may also have been used for this purpose, it is difficult to trace them in the archaeological record. Our experiment of soaking an elk mandible in water proved to be a time-consuming one during the cold part of the year. In warmer conditions and with the help of bacteria and insects, decomposition could proceed quicker. It is challenging to evaluate the effect of the frost that occurred during our experiment and whether it facilitated or delayed the tooth extraction. Considering these technological factors and the required soaking time—more than 5 weeks in our experiment and 4–5 weeks recorded in a previous study by Rainio and Tamboer (2018)—soaking still seems a plausible method. Soaking time will, however, depend on variables such as temperature and environmental conditions. It is also possible that teeth were removed after a shorter soaking time using tools such as hammerstones for percussion or flint blades to cut away the soft tissues around the teeth and bone.

The second successful method (E6) uses a ceramic vessel to hold the water and the mandible, which is then simmered for an extended period. A key limitation of this method, however, is that it requires the use of ceramic technology that only became available towards the middle phases of use of the Zvejnieki cemetery. The use of the wet cooking/ceramic vessel method therefore does not explain the many hundreds of tooth pendants made at Zvejnieki before this time (Macāne 2022). Organic containers made from animal hides and stomachs have been demonstrated to be capable of simmering foodstuffs at low temperatures over longer periods of time (Langley et al. 2023). Mandibles could technically have been simmered using such organic container technologies at Zvejnieki. However, the practicality of these methods remains questionable, since animal heads can be heavy and organic containers may break under their weight or when hot stones and water are added. Furthermore, due to the size and morphology of the elk and wild boar mandibles, it would have been challenging to cook them in both ceramic and soft animal material containers, and it may have been necessary to divide them into smaller pieces. Such a process would likely impact the usability of raw materials obtained from the mandible for osseous tools, as well as the preservation of any edible parts.

While soaking (E4) and wet cooking (E6) do not leave significant traces on the teeth, some soft tissues remain on the tooth roots, requiring additional cleaning and likely resulting in scraping traces on the tooth surface. In fact, periodontal ligaments can occasionally remain attached to the tooth root even after five months of soaking. Thus, it cannot be fully excluded that the “grinding” traces seen on some teeth at Zvejnieki may result from the removal of soft tissues during the extraction and teeth cleaning phase. Future experimental work focusing on replicating these traces would be useful. However, grinding can also be a preparatory stage to enable further modification of the tooth, e.g., by drilling. In sum, while it is technically possible that soaking and boiling methods were used, albeit with some adaptations (such as organic containers), the earth oven method is the only one that does not have any technological or material culture limitations (E7).

The earth oven or cooking pit method allowed the extraction of teeth without any macroscopically observed damage or modification. Teeth were easily extracted from whole animal heads and dislocated mandibles that had been placed in the earth oven. In fact, they practically fell out with minimal encouragement. A key finding was that the extraction of teeth needs to be carried out whilst the teeth and jawbone are still warm, otherwise the gingival cuff, which consists mainly of collagen fibres (Koller and Sapra 2024), will solidify again and effectively “glue” the teeth to the gums/alveoli. As expected, this happened more quickly during the winter experiment when air temperatures were much lower, resulting in more rapid cooling and thus creating a shorter window for tooth extraction.

Practicality, the lack of tooth damage, the ability to extract several teeth relatively quickly and easily, and the recovery of complete sets of teeth are just some of the advantages of using the steaming/earth oven method. Another notable aspect of this method is that there was no need to skin or separate the mandibles in advance. Moreover, the cooking pit would have offered a convenient way to integrate tasks: extracting animal teeth alongside preparing meals (see Domínguez-Solera 2018; Morrison et al. 2022). The complete heads steam-cooked in the two earth oven experiments produced thoroughly cooked meat and brains ready for consumption, teeth that could be easily extracted for pendants and other purposes, and bone material that remained undamaged—at least to the naked eye. The latter could then be utilised for tools, etc.

Earth oven cooking also allows for the processing of larger quantities of food, opening up opportunities for social interaction (see e.g., Yellen 1977a, b; Tuechler et al. 2014; Morisson et al. 2022; Richardson 2024). Feasting and extracting teeth, perhaps in connection with a ceremonial event such as a funeral, may have taken place at Zvejnieki. Evidence of hearths and depressions dug into the natural soil and filled with sooty soil and/or stones, occasionally with burnt fish and animal bones or flint or quartz artefacts, have been documented between the burials (Zagorskis 1970). Elsewhere in northern Europe, earth ovens/cooking pits used by Mesolithic and Neolithic hunter-gatherers have been documented (e.g., Bergman 2008). Similarly, the consumption of food in connection with burial rituals is a widely recorded phenomena in this region/time period (e.g., Brinker et al. 2020; Larsson 1988; Mannermaa 2016). Although a direct link between feasting, funerary activities, and the extraction and immediate use of teeth cannot be proven, we should not rule it out either.

Combining feasting with the performance of other death rituals, such as the making of animal teeth-decorated shrouds/wraps to contain the dead, would not only have practical advantages, such as easy extraction and the possibility of recovering complete sets of teeth, but would also tether domestic and mortuary spheres, uniting the living with the dead. It has been observed (Macāne 2022) that several of the burials that contained evidence of wrapping (Nilson Stutz 2006) also had a higher number of unworked teeth or teeth with grinding traces at the root (occasionally also on the crowns), often complete sets from the same animal. It is possible that some of these were in fact appliqué, sewn onto the wrappings for the deceased. Our experiments showed that complete sets of teeth can be easily achieved by placing entire heads in earth ovens, although other methods cannot be ruled out.

The combined knowledge from our experiments, the practical considerations of the various tooth extraction methods, and the large quantities of animal teeth in some burials at Zvejnieki (exceeding several dozens or hundreds) suggest that the collection of animal teeth for pendant production likely occurred gradually over time. While several animals may have been available during a single extraction event, it seems unlikely that, for example, the 332 teeth of several animal species and dozens of individual animals found in burial 122–123 (see Fig. 4) at Zvejnieki could have been sourced during a short period immediately before the burial. The acquisition of the teeth from at least 24 wild boars, 17 red deer and many other animals must have taken a longer time. The teeth extracted from various animal species or individual animals were probably curated by the community over time and kept for specific occasions. Different stages of macrowear visible on the pendants and unmodified teeth in the same burials suggest different strategies for tooth accumulation (e.g., Larsson 2006; Osipowicz et al. 2023). Teeth were probably gradually incorporated into the clothing and ornaments of certain community members during specific life events. Additionally, teeth pendants could have been acquired through other means, such as reusing curated or older pendants, possibly passed down from other family or community members. All this demonstrates that significant attention was given to the different stages in the life cycle of animal teeth and their use as pendants and personal adornments.

### Animal teeth extraction as part of human-animal interactions

The significance of interaction with the animal has been largely absent from earlier studies focusing on Stone Age pendant production. Therefore, we are missing not only essential parts of the early stages of *chaîne opératoire* of tooth pendant production, but also the human-animal interactions that this stage necessitates. Understanding human-animal interactions, animal processing and tooth extraction practices within hunter-gatherer societies requires the examination of how these relationships were integrated into broader lifeways, including animal-based subsistence economies and craft practices. On the one hand, zooarchaeological studies have primarily focused on taxonomic composition and economic perspectives, emphasising the contents of faunal assemblages or the value of animals as food or raw materials (e.g., Lõugas 2006, 2017; Magnell 2006; Storå 2001). On the other hand, technological aspects and cultural symbolism have dominated the studies of animal teeth as personal ornaments (e.g., Larsson 2006, 2012, 2020; Rigaud 2013; Zagorska and Lõugas 2000). However, these perspectives have not yet fully addressed the extraction process, which, according to our research, involves intensive human-animal interaction. Consequently, we have so far overlooked important potential intersections, such as the acquisition/preparation of food and raw materials and how these activities link to concepts of appropriate treatment of animals and their elements.

Posthumanist and multi-species perspectives, which see teeth pendants as part of complex human-animal relationships within the hunter-gatherer worldviews, place great emphasis on their animal qualities (Conneller 2011; Macāne 2022; Živaljević 2015). Furthermore, the ethnographic evidence from the Khanty highlights the importance of proper treatment of animals, including hunting, carcass processing and food preparation (Jordan 2003; Willerslev 2007). Our experiments, in which the cooking of animals in earth oven could have been part of food consumption, raw material acquisition, and the preparation/decoration of the dead for the burial, highlights how interconnected subsistence, craft, and funerary customs may have been. Such intersections provide excellent opportunities to employ empirical datasets to explore the complexity of human-animal relationships in the life and deathways of hunter-gatherers. The processing of animals and the extraction of materials, such as teeth, were likely intertwined within both the life cycles of the animals and the seasonal activities of humans (Kelly 2010; Cannon 2018). Seasons, weather and environmental conditions would have affected the availability of animal species and consequently impacted hunting methods. All these factors would have influenced the quantity of teeth available for extraction and possibly also the extraction method(s) employed.

The intentional selection of teeth, for example canines of carnivores and incisors of ungulates, or teeth of adult individuals instead of deciduous teeth, indicates deliberate choices that reflect not only the morphological or aesthetic characteristics of the teeth of different animals but also the importance of specific species within the hunter-gatherer’s cosmological worldviews. The methods of tooth extraction may also have expressed relationships with specific animal species or individual animals, reflecting varying cultural beliefs or meanings. It is possible that beliefs or taboos were not only attached at the species or individual level, but were associated with different body parts and affected their preparation or processing (e.g., Reitz and Wing 2008; Živaljević 2015; Dominguez-Solera 2018). Animal agency may therefore have been expressed through selected extraction methods, some of which facilitated the maintenance of the animal’s integrity, while others required more intrusive interaction and destruction. Viewed in this light, the cooking pit may have been perceived as a more respectful treatment of the animal, whereby teeth were “given by the animal” with ease (Jordan 2003).

The results of our experiments point to possible differences between teeth extraction methods applied to younger animals in comparison to older animals — tooth extraction from the latter proves to be significantly more challenging. The age of the animal also affects the aesthetics of the teeth, since there are differences between deciduous and fully grown teeth. The aesthetic preferences, as well as selective hunting of certain age groups of some species, may in part account for why a larger number of teeth from adult animals are found in the Zvejnieki assemblages. More experimental work is needed to test a wider variety of teeth from different animal species (including human) and of different ages to more fully explore the limitations and potential of hunter-gatherer extraction processes.

## Conclusion

This study highlights the fundamental role of animal tooth extraction in the production of personal ornaments within hunter-gatherer societies. Previous studies have neglected this key aspect in the technological *chaîne opératoire* of animal teeth pendants. This knowledge gap has arguably led to the assumption that teeth used for pendant making were readily available as pre-forms, requiring little time/energy investment in the sourcing and extracting phase. We tested seven different methods for extracting animal teeth. While some methods, such as air-drying and direct heating of mandibles, were unsuccessful, others, like cutting and percussion, yielded positive results but caused damage to the teeth and bones. Three methods successfully provided undamaged teeth, ready for further modification. Our experiments suggest that tooth extraction was not only a functional aspect of processing animal carcasses, but also deeply embedded in everyday life, notably the cooking practices of the pendant-making communities. A key finding from the cooking experiments (wet cooking and steaming in an earth oven) is that tooth extraction must occur within a short window of time after the mandible is removed from the pot or oven, while the teeth, gingival cuff, and surrounding bone are still warm. It is at this stage that the periodontal ligament and gingival cuff have gelatinized. If the extraction is delayed, the collagen cools and solidifies, effectively glueing the teeth in place, making them impossible to remove manually. This solidification process occurred more rapidly, as might be expected, during the colder winter experiments.

While this study focused principally on the tooth pendant assemblage from the Zvejnieki cemetery, our results have broader implications for understanding tooth extraction and pendant production across prehistory. By examining techniques used for tooth extraction, we have gained valuable insights into human behaviour and cultural practices during the Stone Age. However, significant gaps remain in our understanding of this process. Future research should focus on the detailed examination of teeth for visible traces of extraction to further elucidate this early stage in the *chaîne opératoire.* Traces left by extraction methods would need a comparison with traces produced on teeth by subsistence-related butchery activities, specifically the removal of the tongue. The extraction of human teeth, as well as the teeth of dogs and other carnivores, as well as molars, was not addressed in our study — all these need to be examined in detail in the future. We further hope this research will encourage scholars in the fields of material culture and zooarchaeology to rethink and co-investigate the often-overlooked steps in the *chaîne opératoire* of pendant production, shedding a critical light on the complexity and significance of these practices. By addressing these gaps, we can enrich our understanding of personal ornament production and more fully explore the diverse contexts of human-animal relationships in prehistoric societies. A better understanding of the extraction process can provide more comprehensive insights into the life histories of teeth pendants: from the point of animal capture, death and processing, through the removal of the teeth to their possible modification (suspension), their wear from use, and finally their deposition.

## Supplementary Information

Below is the link to the electronic supplementary material.

Supplementary file1 (DOCX 24 KB)

## Data Availability

No datasets were generated or analysed during the current study.

## Declarations

### Geographical coordinates

Zvejnieki cemetery (archaeological site) 57.775387, 25.229457. Data collected by authors using Google Maps 24.09.2024.
