# Supplementary material for: First stage in technological production of Stone Age animal teeth pendants: evidence from Zvejnieki (Latvia) and wider social implications
Source: Archaeol Anthropol Sci. 2025 Jun 20;17(7):148. doi: 10.1007/s12520-025-02260-0 (PMC12181117; doi:10.1007/s12520-025-02260-0)
Supplement: Supplementary file 1 — Supplementary file1 (DOCX 24 KB) [file 12520_2025_2260_MOESM1_ESM.docx]

# **Supplementary information (SI)**

**Detailed description of experiments**

### **E1: Cutting**

***Experiment description and results***

| **Specimen** | **State** | **Used in previous experiment** | **Tool used** | **Working time** | **Outcome** |
| --- | --- | --- | --- | --- | --- |
| AN6: elk mandible, juvenile | Frozen | No | Flint blade #7, #8 | #7: 25 mins  #8: 15 mins  Total: 40 mins | Success: tooth extracted |
| AN4: elk mandible, juvenile | Frozen | Direct heat/fire | Flint blade #3 | 15 mins | Failure: tooth not extracted |
| AN3: elk mandible, adult | Frozen | Direct heat/fire | Flint blade #1, #2 | #1: 2 mins  #2: 2 mins  Total: 4 mins | Failure: tooth not extracted |
| AN1: boar mandible, adult | Fresh | Air-drying | Flint blade # 5, #6 | # 5: 3mins  #6: 5 mins  Total: 8 mins | Failure: tooth not extracted |

Table S.I.1. Results of cutting experiments detailing animal parts and tools used, duration of use, and outcome.

**EM2: Percussion**

***Experiment description and results***

| **Specimen** | **State** | **Used in previous experiment** | **Tool used** | **Working time** | **Outcome** |
| --- | --- | --- | --- | --- | --- |
| AN11: elk mandible, adult | Fresh | No | Angular, rounded stone | 8 minutes | Success: tooth extracted, but root broken and soft tissues still attached to the root |
| AN10: elk mandible, adult | Fresh | No | Angular stone | ca. 2 minutes | Success: tooth extracted, but broken root and soft tissues still on root |
| AN2: boar mandible, adult | Frozen | Cooking pit | Rounded stone    Antler tine, hammerstone | 3 minutes    4 minutes | Failure: tooth not extracted  Success: tooth extracted |
| AN1: boar mandible, adult | Fresh | Air-drying | Wooden stick  Rounded stone | 5 minutes  3 minutes | Failure: tooth not extracted |

Table S.I.2. Results of percussion experiments detailing animal parts, their condition, and previous uses, tools used and duration of use, and outcome.

###

### **E4: Soaking**

***Experiment description and results***

| **Time** | **Interventions and observations** |
| --- | --- |
| 0 days | Elk mandible (skinned) placed in the bucket with water from the pond. |
| 8 days | First extraction attempt – teeth are not moving; gingival cuff is strongly holding the teeth. |
| 15 days | The teeth move slightly, but are still firmly held in place by the gingival cuff. The soft tissues are white and beginning to rot. |
| 28 days | The teeth move slightly but are still firmly held in the gingival cuff. Soft tissues are decaying, and worms can be seen in the cavities above the water line. The decayed soft tissue has been removed, but the area around the front teeth and the gingival cuff is firmly anchored and cannot be removed. |
| 36 days | The teeth are moving in the alveoli, and the bone feels softer, but the gingival cuff still firmly holds the teeth in place. Soft tissues have rotten away and have been removed, and mandible is clean, except for the distal part, area around the mandibular symphysis and front teeth, where soft tissues are remaining, and the gingival cuff are strongly attached to the mandible. |
| 24 weeks | The incisors were extracted from the mandible by pulling them out. Some teeth still had the periodontal ligament attached, while others were completely clean. |

Table S.I.3. Results of a soaking experiment detailing interventions, observations, duration of soaking, and outcome.

###

### **E5: Direct heat/fire**

***Experiment description and results***

| **Time** | **Interventions and observations** |
| --- | --- |
| O minutes | AN3 and AN4 were placed next to the fire (heavy wind). |
| 10 minutes | Elk (AN 3) start sizzling. |
| 25 minutes | The mandibles were moved further away from the fire (approximately 30 cm) as the heat was too intense and burned the soft tissue and skin on the mandibles. |
| 30 minutes | The elk mandible (AN4) became charred. During the first tooth extraction attempt for both AN3 and AN4, the incisors did not move. The lip and skin protected the teeth from fire and heat. |
| 120 minutes | In the subsequent attempt to extract teeth from AN4, the fourth incisor was found to be brittle, and its crown broke during the extraction attempt. The lip was then removed. |
| 130 minutes | The elk mandible was repositioned near the fire to heat the bone. The proximal part of the mandible was pointed toward the fire to protect the teeth from excessive heat. The mandible was then divided into halves, resulting in the eruption of the first permanent incisors from the left side, and the crown of the second permanent left incisor dislodged from the alveoli. |
| 140 minutes | AN3 was removed from the fire for another extraction attempt, but the incisors remained immobile. The charred soft tissues and meat were cleaned away. A flint blade (#1) was used to cut out the incisors, but the mandible was very hard, and four minutes of cutting yielded no results. The mandible was placed back near the fire with the proximal part facing the flames to keep the incisors away from the heat. |
| 200 minutes | Subsequent attempts to extract the teeth by pulling them out were unsuccessful. During one extraction attempt, the third left incisor of AN3 broke. The horizontal lines were visible on the tooth enamel, most likely caused by the heat. The experiment was then terminated. |

Table S.I.4. Results of direct heat/fire experiments, detailing interventions and observations during the experiment.

###

### **E6: Cooking - boiling (wet/ceramic)**

***Experiment description and results***

| **Time** | **Interventions and observations** |
| --- | --- |
| 0 minutes | A fire was lit next to the pottery vessel to prepare charcoal, which was then placed around the pot. The pot was positioned approximately 40 cm away from the fire and filled halfway with water at approximately +15°C. A roe deer mandible (AN7) with skin was placed into the pot (the remaining head was used in the cooking pit) next to the fire. At this stage, no hot embers were placed around the pot. |
| 40 minutes | Then, some hot embers were placed around the pot. According to the thermocouple, the temperature of the embers varied between 300 and 525 °C. The temperature in the center and on the outside of the pot was 170 °C, while in the lower part it was 220 °C. The water temperature in the pot was 45 °C. |
| 85 minutes | More hot embers were transferred from the nearby fire to the pot. The water temperature immediately rose from 47 °C to 65–67 °C. The temperature of the embers around the pot fluctuated between 400 and 600 °C. |
| 115 minutes | Additional hot embers were placed around the pot. The water temperature was 64°C, while the embers around the pot ranged from 250–550°C, depending on the side. The temperature of the lower part of the pot (outside) was 220°C. |
| 175 minutes | More hot embers were added around the pot, raising the water temperature to 51°C. |
| 188 minutes | The first attempt to extract the teeth was made, but they remained immobile. The water temperature was 66°C. |
| 213 minutes | The water temperature reached 72°C when larger pieces of embers were placed around the pot. |
| 220 minutes | With larger embers around the pot, the water temperature rose immediately to 80°C. |
| 245 minutes | More cold water was added to the pot, as half of the water had evaporated. |
| 320 minutes | The water temperature reached 88°C. Larger pieces of burning wood (with flames) were added around the pot. |
| 330 minutes | After larger pieces of burning wood were placed around the pot, the water began to boil and foam. |
| 345 minutes | The water was boiling violently, and more cold water was added to the pot. |
| 360 minutes | Water t° is 100 °C. |
| 395 minutes | Water t° is 95 °C. |
| 430 minutes | The water temperature was 75°C when the roe deer mandible was taken out. The skin and soft tissues from the mandible were easily removed, but the incisors remained firmly in the alveoli. |
|  | The experiment was terminated for the day, and the pot was left in the fireplace to cool down slowly overnight. The roe deer mandible was taken out and placed in a safe location to protect it from wild and domestic animals. The experiment resumed the next morning. |
| 0 minutes | The ceramic pot was taken into use again and placed next to fire. The roe deer mandible was further cleaned of the remaining soft tissues (cooked the previous day) to expedite the boiling process and was then placed back into the pot. The water temperature in the pot was 19°C, the same as the ambient air temperature. |
| 95 minutes | Water t° is 90°C in the water. |
| 115 minutes | The roe deer mandible was removed from the pot, and the teeth (incisors) could be easily pulled out of the mandible. |

Table S.I.5. Results of direct heat/fire experiments, detailing interventions and observations during the experiment.

###

### **E7: Cooking pit**

***Experiment description and results***

Winter experiment 26.-27.02.2022.

| **Time** | **Interventions and observations** |
| --- | --- |
| O minutes | A fire was lit on the stones in the pit using wet broadleaf wood, primarily alder and birch, with birch bark used to ignite the fire. |
| 240 minutes | The wild boar hide was placed over the hot embers for insulation, with the fur side down and the fat side up, providing a surface for the placement of the animal heads. The head of the wild boar (AN2) and mandible of the elk (AN5), along with the wild boar feet, were positioned on the wild boar skin and covered with snow. The other half of the skin was used to cover the heads. A thin layer of sand, approximately 5 cm thick, was then spread over the skin. |
| 245 minutes | A new fire was started above the heads and burned for 2.5 hours. |
| 405 minutes | The hot embers were covered with turf and a thin layer of sand for the night. |
| 1050 minutes | When the pit was reopened, the temperature inside remained very high. The wild boar head and elk mandible were removed from the pit and placed on the snow. They were thoroughly cooked, and the insulation provided by the wild boar skin and the snow had evidently facilitated the steaming/cooking process. |
| 1065 minutes | The tooth extraction process commenced once the heads had cooled sufficiently to be handled. The incisors were easily extracted by pulling them out, and they were completely free of any soft tissues. |

Table S.I.6. Results of cooking pit experiments held during winter, detailing interventions and observations during the experiment.

Summer experiment, 30.-31.07.2022.

| **Time** | **Interventions and observations** |
| --- | --- |
| O minutes | A new fire was started after the first one was destroyed by the thermocouple. |
| 65 minutes | Thermocouple (bottom of the pit) 560° C, on top of fire 650° C. |
| 95 minutes | Thermocouple (top and bottom of the pit) 500°C. |
| 215 minutes | Thermocouple (bottom of the pit) 400° C, on top of fire 670° C. |
| 250 minutes | The temperature at the bottom of the pit was recorded at 420°C, while the temperature on top of the fire was 700°C. As the fire began to die down, the hide of a roe deer was placed on the hot embers. The heads of the roe deer (AN7 and AN8) were placed on the skin with a splash of water, along with two fish positioned in the northern part of the pit. All items were covered with a thick layer of ferns. The thermocouple was placed above the heads, within the ferns, and the soil was then filled back over the ferns. Immediately after covering the pit with soil, the temperature was 380°C at the bottom of the pit and 20°C on top of the heads. |
| 275 minutes | A new fire was lit above the heads, on the soil. The thermocouple recorded an upper temperature of 32°C and a lower temperature of 345°C. |
| 295 minutes | Thermocouple: upper t° is 27°C, lower t° is 320°C. |
| 330 minutes | Thermocouple: upper t° is 58°C, lower t° is 280°C. |
| 355 minutes | Thermocouple: upper t° is 56°C, lower t° is 265°C. |
| 395 minutes | Thermocouple: upper t° is 60°C, lower t° is 235°C. |
| 450 minutes | Thermocouple: upper t° is 70°C, lower t° is 212°C. |
| 465 minutes | Once the fire had burned out, the hot embers were covered with turf and left overnight. The thermocouple readings were 74°C for the upper temperature and 213°C for the lower temperature. |
| 1115 minutes | Thermocouple: upper t° is 81°C, lower t° is 96°C. |
| 1215 minutes | Thermocouple: upper t° is 75°C, lower t° is 82°C. |
| 1220 minutes | By morning, the thermocouple recorded an upper temperature of 66°C and a lower temperature of 77°C. The cooking pit was then opened, and the upper thermocouple was removed. The roe deer heads were taken out and placed on the ground. After 10-15 minutes, the extraction process began. The incisors of roe deer (AN8) were easily pulled out, as the mandible had divided into left and right sides when detached from the head. The head was cleaned of skin and soft tissues, which were removed very easily. |

Table S.I.7. Results of cooking pit experiments held during summer, detailing interventions and observations during the experiment.
